# Prenylcysteine oxidase 1, an emerging player in atherosclerosis

**DOI:** 10.1038/s42003-021-02630-z

**Published:** 2021-09-21

**Authors:** C. Banfi, R. Baetta, S. S. Barbieri, M. Brioschi, A. Guarino, S. Ghilardi, L. Sandrini, S. Eligini, G. Polvani, O. Bergman, P. Eriksson, E. Tremoli

**Affiliations:** 1 Centro Cardiologico Monzino IRCCS, Milano, Italy; 2 Cardiovascular Tissue Bank of Milan, Centro Cardiologico Monzino IRCCS, Milano, Italy; 3 Department of Clinical Sciences and Community Health, Cardiovascular Section, University of Milan, Milano, Italy; 4 Department of Cardiovascular Disease, Development and Innovation Cardiac Surgery Unit, Centro Cardiologico Monzino IRCCS, Milano, Italy; 5 Department of Medicine Solna, Karolinska University Hospital, Karolinska Institutet, Stockholm, Sweden

**Keywords:** Mechanisms of disease, Atherosclerosis

## Abstract

The research into the pathophysiology of atherosclerosis has considerably increased our understanding of the disease complexity, but still many questions remain unanswered, both mechanistically and pharmacologically. Here, we provided evidence that the pro-oxidant enzyme Prenylcysteine Oxidase 1 (PCYOX1), in the human atherosclerotic lesions, is both synthesized locally and transported within the subintimal space by proatherogenic lipoproteins accumulating in the arterial wall during atherogenesis. Further, Pcyox1 deficiency in Apoe^-/-^ mice retards atheroprogression, is associated with decreased features of lesion vulnerability and lower levels of lipid peroxidation, reduces plasma lipid levels and inflammation. PCYOX1 silencing in vitro affects the cellular proteome by influencing multiple functions related to inflammation, oxidative stress, and platelet adhesion. Collectively, these findings identify the pro-oxidant enzyme PCYOX1 as an emerging player in atherogenesis and, therefore, understanding the biology and mechanisms of all functions of this unique enzyme is likely to provide additional therapeutic opportunities in addressing atherosclerosis.

## Abstract (web summary)

Banfi et al. investigate the role of Prenylcysteine oxidase 1 (PCYOX1), an enzyme implicated in catabolism of prenylated cysteine, in human and murine atherosclerosis. They find that loss of PCYOX1 is anti-atherogenic and understanding its biology and functions can potentially provide significant therapeutic opportunities in addressing atherosclerosis.

## Introduction

Atherosclerotic cardiovascular disease (CVD) remains a leading cause of vascular disease worldwide and a public health challenge^1^. Even with access to the highest technology and most recently available secondary prevention therapies, the burden of recurrent cardiovascular events remains unacceptable, despite optimal treatment with contemporary intervention and pharmacologic agents^1,2^.

In the meantime, research has continued to provide new insights into the pathophysiology of atherosclerosis and the mechanisms of its clinical complications. These advances have considerably increased our understanding of the complexity of atherosclerosis, allowing the development of new therapeutic approaches to combat CVDs^3^, but still many questions remain unanswered^4^. In the field of lipids, for example, albeit there is solid experimental evidence that low-density lipoprotein (LDL) particles are atherogenic, the exact mechanisms by which they drive the atherosclerotic process from beginning to end remains unsettled^5^. Moreover, although many decades of research have supported the concept that oxidized LDL particles can promote atherogenesis, the “oxidative theory of atherosclerosis” has not yielded an actionable therapy^3^. Furthermore, contrary to the long-standing belief that high-density lipoprotein (HDL) particles are anti-atherogenic, the results of recent genetic association studies, as well as large clinical trials targeting increases in HDL cholesterol to reduce CVD risk, do not support a general protective role for these lipoproteins against atherosclerosis. Such observations have contributed to the emerging concept that the quality, rather than the quantity, of HDL plays a central role in CVD risk protection^6,7^, thus leaving the field wide open in better understanding the complexities of lipoprotein biology and their roles in healthy and disease conditions^8^.

Recent proteomic studies have made an important contribution to this issue, revealing a previously unappreciated complexity of plasma lipoproteins. These studies have extended the number of lipoprotein-associated proteins and suggested that LDL and HDL subclasses have unique profiles that may modulate specific functions beyond lipid metabolism^9^. The findings that abnormalities of many apolipoproteins and multiple lipoprotein-associated proteins are related to atherosclerosis burden have introduced the concept that functionality could be more strongly linked to cardiovascular risk than lipoprotein quantity^6^.

Using a proteomic approach we found, in human lipoproteins, the presence of prenylcysteine oxidase (PCYOX1)^10^, a protein whose biological functions, since its discovery in 1997^11^, have been exclusively restricted to the metabolism of prenylated proteins^12^. The basic features of the chemical mechanism through which PCYOX1 catalyzes the degradation of prenylcysteine during the normal turnover of prenylated proteins have been previously described^13^. Basically, PCYOX1 was described as a Flavin adenine dinucleotide (FAD)-dependent thioether oxidase that generates a stoichiometric amount of hydrogen peroxide (H_2_O_2_). Indeed, we showed that PCYOX1 bound to the apoB100-containing lipoproteins can generate H_2_O_2_ in vitro^10^. The incubation of human lipoproteins with the PCYOX1 substrate, farnesylcysteine (FC)^13^, results in a progressive generation of H_2_O_2_ due to the enzyme activity, which is more marked with very low-density lipoprotein (VLDL) than with LDL or HDL, reflecting the different PCYOX1 content in the lipoprotein classes^10^. Furthermore, we previously demonstrated that the hepatocyte cell line HepG2, a well-known model for the study of lipoprotein biogenesis^14^, synthesizes and secretes PCYOX1 in association with lipoproteins^10^.

Thus, the findings that PCYOX1 represents a novel lipoprotein-associated protein, which might contribute to the oxidation of the lipoproteins through the generation of the reactive oxygen species (ROS) H_2_O_2_, prompted us to investigate the biological role of this unique enzyme in the development of atherosclerosis.

## Results

### PCYOX1 associated with human atherogenic apoB100-containing lipoproteins generates H_2_O_2_, which in turn induces lipoprotein oxidation in vitro

**Fig. 1:**
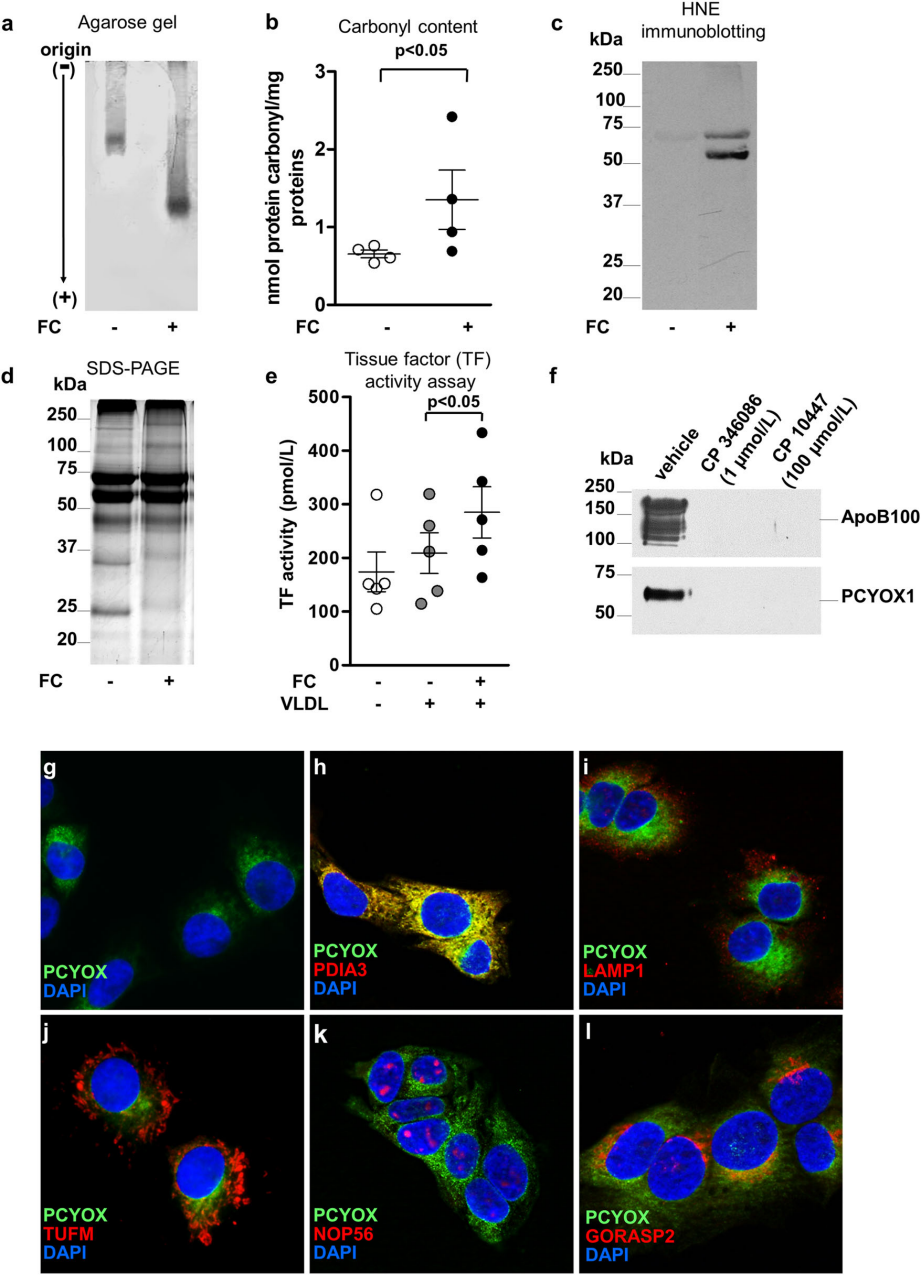
PCYOX1 bound to human atherogenic apoB100-containing lipoproteins generates H_2_O_2_, which in turn induces lipoprotein oxidation in vitro, and associates to nascent apoB100-containing lipoproteins in the endoplasmic reticulum. Agarose gel electrophoresis (**a**), carbonyl content (**b**), 4-hydroxy-2-nonenal (HNE) immunoblotting (**c**), and SDS-polyacrylamide gel (**d**) of human VLDL incubated with FC (200 µmol/L) for 72 h. **e** Tissue factor (TF) activity in HAEC incubated with FC-treated VLDL. *p* < 0.05 vs vehicle-treated VLDL, by Student’s *t* test and ANOVA, respectively. Data in **b**, **e** are presented as circle plot, with each circle representing an individual sample and bars showing the mean value ± SEM of 4 and 5 independent experiments, respectively. **f** PCYOX1 and apoB100 immunoreactivity of apoB100-containing lipoproteins isolated from HepG2 cells incubated with vehicle or MTP inhibitors, CP346086 and CP10447. **g**–**l** Immunofluorescence of PCYOX1 (green) (**g**) in HepG2 cells and co-staining (red) with antibodies against PDIA3 (**h**), LAMP1 (**i**), TUFM (**j**), NOP56 (**k**), and GORASP2 (**l**). Nuclear reference DAPI in blue, objective ×40.

As shown Fig. 1, incubation of the apoB100-containing lipoprotein class that has the highest PCYOX1 levels (VLDL) with PCYOX1 substrate FC led to an increased lipoprotein electrophoretic mobility on agarose gel, an increase in the carbonyl content, a greater immunoreactivity for the lipid peroxidation-derived aldehyde 4-hydroxy-2-nonenal (HNE), and an extensive non-proteolytic scission of apolipoproteins^15^ (Fig. 1a–d), which together suggests that PCYOX1 bound to lipoproteins can generate H_2_O_2_. The procoagulant activity of tissue factor (TF), a gene modulated by oxidant species^16,17^, was significantly increased in human aortic endothelial cells (HAECs) incubated with FC-treated lipoproteins (Fig. 1e). Further, human differentiated macrophages uptake FC-treated LDL and present a larger cell size in comparison to the relatively smaller macrophages incubated with the vehicle-treated LDL (Supplementary Fig. 1a, b).

### PCYOX1 associates with nascent apoB100-containing lipoproteins in the endoplasmic reticulum (ER)

Furthermore, the inhibition of microsomal triglyceride transfer protein completely prevented the release of PCYOX1 bound to apoB100-enriched lipoprotein (Fig. 1f), indicating that, at least in vitro, PCYOX1 binds to the lipoproteins during their biogenesis in the lumen of ER^18^. In accordance with this hypothesis, by immunostaining with reference markers for different subcellular organelles (identified by the Human Protein Atlas project, https://www.proteinatlas.org/), we found that, in HepG2 cells, PCYOX1 co-localizes with protein disulfide-isomerase A3 (PDIA3), a marker for ER (Fig. 1g, h). In contrast, we did not observe co-localization of PCYOX1 with lysosome-associated membrane glycoprotein 1 (LAMP1), a marker for lysosomes; with elongation factor Tu, mitochondrial (TUFM), a marker for mitochondria; and with nucleolar protein 56 (NOP56), a marker for nucleoli. The limited colocalization with Golgi (Golgi reassembly-stacking protein 2 (GORASP2)) is expected as the protein travels through the secretory pathway (Fig. 1i–l).

### PCYOX1 silencing affects the cellular proteome by influencing multiple functions

**Fig. 2:**
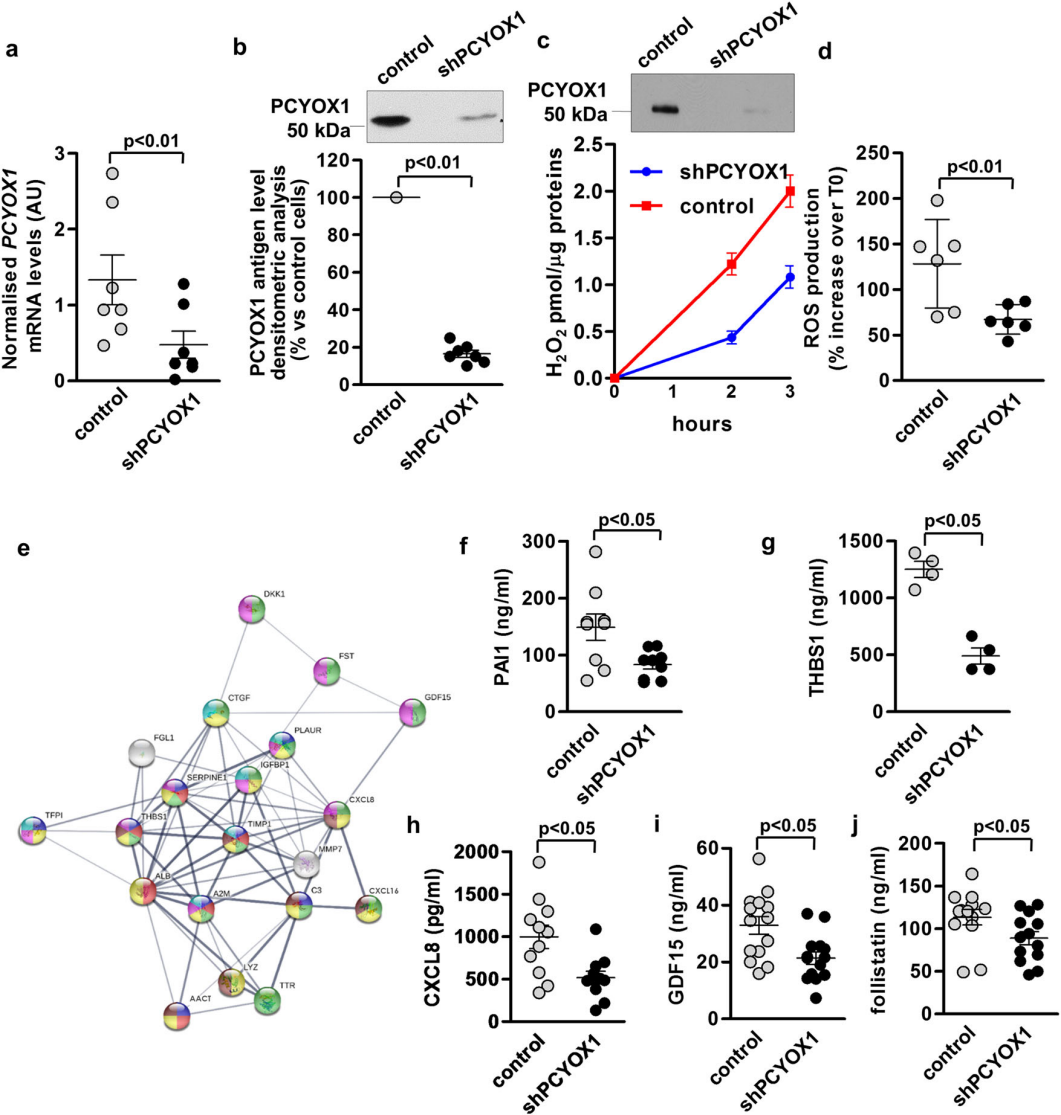
PCYOX1 silencing affects the cellular proteome. *PCYOX1* mRNA normalized to the housekeeping gene *18S* rRNA (**a**, *n* = 7) and protein (**b**, *n* = 7) in PCYOX1-silenced HepG2 cells (shPCYOX1). *p* < 0.01 vs control cells by Student’s *t* test. **c** PCYOX1 immunoblotting (upper panel, *n* = 7) and H_2_O_2_ production (lower panel, *n* = 3) of apoB100-containing lipoproteins isolated from control or shPCYOX1 cell conditioned media. Data are expressed as mean value ± SEM of H_2_O_2_ pmol/µg proteins over time. **d** ROS production in PCYOX1-silenced HepG2 cells (*n* = 6). *p* < 0.01 vs control cells by Student’s *t* test. **e** GO analysis of secreted proteins downregulated by PCYOX1 silencing highlighting enriched biological processes: blue, negative regulation of peptidase activity; red, platelet degranulation; green, regulation of signal transduction; yellow, response to stress; violet, negative regulation of response to stimulus; brown, inflammatory response; light blue, response to wounding. **f**–**j** Levels of PAI-1, THBS1, CXCL8, GDF15, and Follistatin measured by ELISA. *n* > 4. *p* < 0.05 vs control cells by Student’s *t* test. Data are presented as circle plot, with each circle representing an individual sample and bars showing the mean value ± SEM.

To explore the biological functions of PCYOX1, we combined a gene silencing approach with quantitative proteomics. In HepG2 cells, PCYOX1 silencing resulted, without any sign of cytotoxicity (Supplementary Fig. 1c–e), in a significant reduction of *PCYOX1* mRNA and protein and of PCYOX1 bound to apoB100-containing lipoproteins (Fig. 2a–c, upper panel). PCYOX1 activity, assessed as H_2_O_2_ generation by apoB100-containing lipoproteins isolated from the media of PCYOX1-silenced HepG2 cells, was lower than that generated by the apoB100-containing lipoproteins derived from control cells (Fig. 2c, lower panel). Further, cellular ROS was also significantly reduced in PCYOX1-silenced HepG2 cells (Fig. 2d). In contrast, PCYOX1 overexpressing CHO cells produced a higher amount of oxidants (Supplementary Fig. 1f–h).

Gene ontology (GO) analysis of proteins that were less secreted after PCYOX1 silencing in HepG2 cells (Supplementary Tables 1 and 2) revealed the enrichment of GO terms related to response to stress (*n* = 14, *p* = 1.13e−05, i.e., plasminogen activator inhibitor 1 (PAI-1), thrombospondin-1 (THBS1), and interleukin-8 (IL-8; C-X-C chemokine motif ligand 8 (CXCL8)), negative regulation of response to stimulus (*n* = 10, *p* = 2.20e−05, i.e., growth differentiation factor 15 (GDF-15), follistatin, and CXCL8), platelet degranulation (*n* = 6, *p* = 3.65e−07, i.e. PAI-1 and THBS1), regulation of signal transduction (*n* = 14, *p* = 5.27e−06, i.e., GDF-15 and follistatin), inflammatory response (*n* = 7, *p* = 1.57e−05, i.e., THBS1), negative regulation of peptidase activity (*n* = 8, *p* = 8.13e−08, i.e., THBS1 and PAI-1), and response to wounding (*n* = 6, *p* = 0.00027, i.e., connective tissue growth factor (CTGF), insulin-like growth factor binding protein-1 (IGFBP1), metalloproteinase inhibitor 1 (TIMP1)) (Fig. 2e and Supplementary Data 1). Enrichment of proteins involved in the regulation of proteolysis (*n* = 11, *p* = 1.85e−07) and defense response (*n* = 12, *p* = 1.0e−06) was observed when upregulated secreted proteins were analyzed (Supplementary Data 1). The lower levels of secreted proteins relevant in platelet activation and inflammation, such as PAI-1, THBS1, CXCL8, GDF-15, and follistatin, were verified in independent experiments by immunoenzymatic assays (Fig. 2f–j). Since thrombospondin and PAI-1 are involved in platelet adhesion^19^, using two in vitro systems including the secretome from PCYOX1-silenced cells, we found that the adhesion of human platelets to fibrinogen-coated plates or human endothelial cells was significantly lower in the presence of the secretome derived from PCYOX1-silenced cells (Supplementary Fig. 1i, j).

### PCYOX1 is highly abundant in human and murine atherosclerotic lesions

**Fig. 3:**
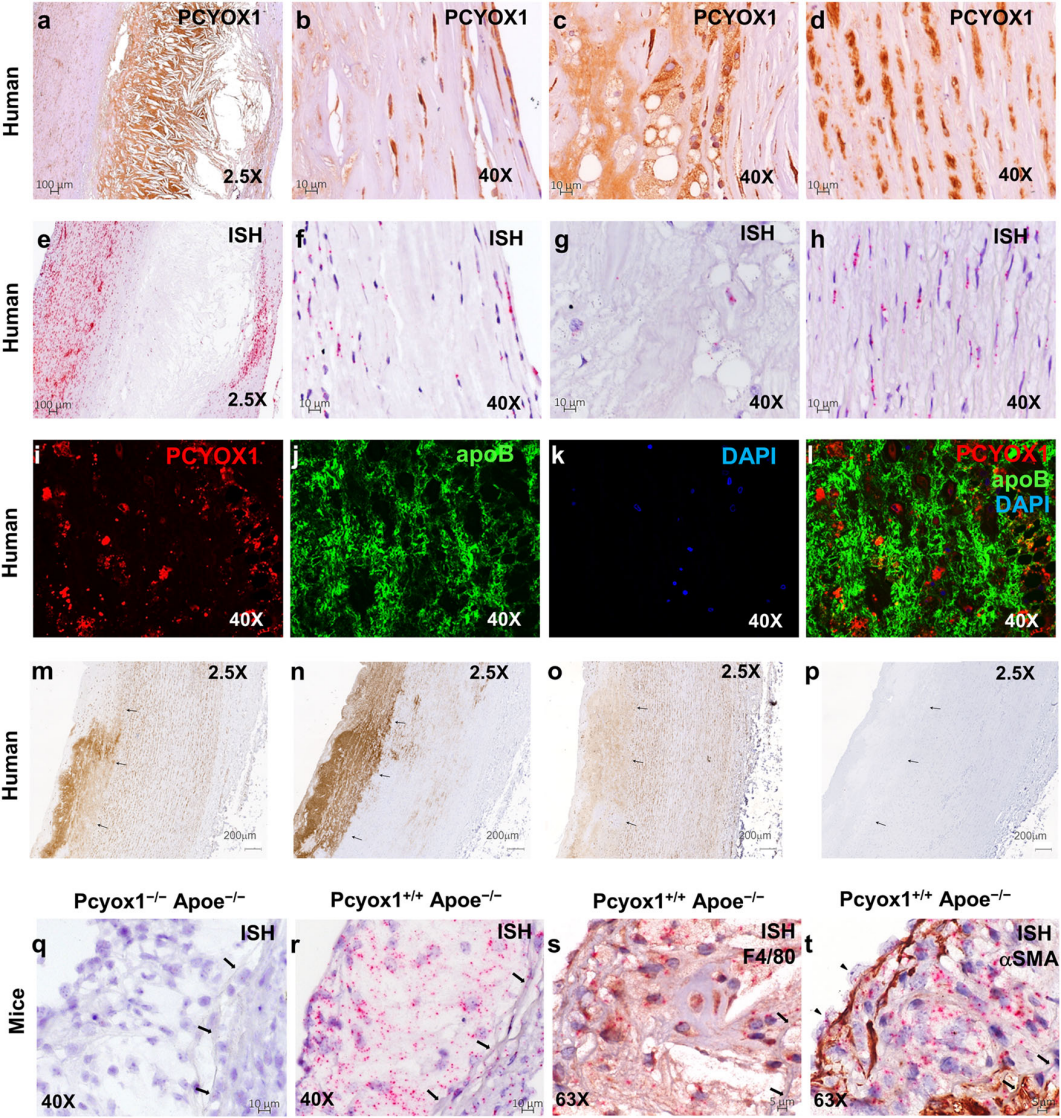
PCYOX1 is abundant in human and Apoe^−/−^ mice atherosclerotic lesions. Sections of human atherosclerotic lesions were subjected to IHC (**a**–**d**) or ISH (**f**–**h**) analysis for the detection of PCYOX1 protein and mRNA, respectively, and counterstained with hematoxylin. (**a**; objective ×2.5; **b**–**d**; objective ×40). **e** Representative image of the positive control probe (housekeeping gene, Ubiquitin C), demonstrating mRNA integrity (objective ×2.5). **f**–**h** *PCYOX1* mRNA staining (red punctate dots, objective ×40). **b**, **f** luminal side; **c**, **g** lipid-rich regions; **d**, **h** tunica media. **i**–**l** PCYOX1 and apoB immunofluorescence in human atherosclerotic lesions counterstained with DAPI (confocal images, objective ×40). Shown are representative images from one donor. **m**–**p** Serial sections immunostained for PCYOX1 (**m**), apoB (**n**), and farnesyl groups deriving from isoprenylated proteins (**o**) or in the absence of primary antibody (negative control, **p**). Arrows indicate the internal elastic lamina. Shown are representative images from another donor. Negative controls are shown in Supplementary Fig. 2. **q**–**t** Aortic root sections from Pcyox1^+/+^/Apoe^−/−^ and Pcyox1^−/−^/Apoe^−/−^ mice fed a HFD for 8 weeks subjected to ISH for the detection of *Pcyox1* mRNA alone (**q**, **r** objective ×40) or in combination with immunostaining for cell-type-specific protein markers (objective ×63), F4/80 for macrophages (**s**), and α-SMA for SMC (**t**). Tissue sections were counterstained with hematoxylin. Arrows in **q**–**s** indicate internal elastic lamina. Arrowheads in **t** indicate the expression of PCYOX1 in luminal ECs.

Given the association of PCYOX1 with apoB100-containing lipoprotein and its potential pro-atherogenic effects, we investigated whether the enzyme is present in human vascular lesions. Formalin-fixed, paraffin-embedded (FFPE) sections of human atherosclerotic aortic tissue samples (ascending aorta and aortic arch) were immunostained with two different primary antibodies recognizing distinct portions of human PCYOX1: a rabbit polyclonal antibody developed and validated by the Human Protein Atlas project (HPA035193) and a mouse monoclonal antibody (mAb) from Santa Cruz Biotechnology (sc-136391). In human vascular lesions, both antibodies showed intense cytosolic staining in intimal cells (Fig. 3a–c) and in medial smooth muscle cells (SMCs) (Fig. 3d). In addition, PCYOX1 immunostaining was widespread in the lipid-rich regions (Fig. 3c) and adjacent to cholesterol clefts, consistent with both an intracellular and extracellular distribution of the protein. In normal arterial tissue, PCYOX1 expression was evident in medial SMCs, but no extracellular deposits could be detected (Supplementary Fig. 2). To validate this issue, FFPE sections of atherosclerotic tissue were interrogated with a probe targeting the 292–1357 region of the human *PCYOX1* mRNA. In agreement with immunohistochemical (IHC) results, in situ hybridization (ISH) analysis revealed the presence of *PCYOX1* mRNA in both intimal (Fig. 3f) and medial cells (Fig. 3h), while it was scarce or nearly absent in the acellular lipid-rich domains (Fig. 3g).

Overall, these findings suggested that PCYOX1 present in atherosclerotic lesions is both synthesized locally and transported within the subintimal space by proatherogenic lipoproteins accumulating in the arterial wall during atherogenesis. To address this hypothesis, we used double immunostaining to visualize PCYOX1 and apoB, the major protein constituent of the atherogenic apoB100-containing lipoproteins, in atherosclerotic lesions. Both confocal immunofluorescence (Fig. 3i–l) and double IHC (Supplementary Fig. 3) showed the presence of extracellular staining for PCYOX1 in intimal regions rich in apoB deposits, supporting the concept of a lipoprotein-mediated influx of PCYOX1 from the circulation. In addition, immunostaining of serial tissue sections for PCYOX1, apoB, and farnesyl groups, deriving from isoprenylated proteins, suggested that farnesyl cysteine, the substrate of PCYOX1 enzymatic activity, is indeed present in these regions (Fig. 3m–p).

Furthermore, we generated a double knockout mouse model (Pcyox1^−/−^/Apoe^−/−^ mice) for studying the role of PCYOX1 in atherosclerosis, and PCYOX1 analysis by IHC and ISH was also performed on aortic root sections of Pcyox1^+/+^/Apoe^−/−^ and Pcyox1^−/−^/Apoe^−/−^ mice on high-fat diet (HFD) for 8 weeks. Consistent with human findings, ISH analysis, performed with an RNAscope target probe designed to be highly specific for a portion of the exon 6 of the murine *Pcyox1* gene (i.e., the exon deleted in Pcyox1^−/−^/Apoe^−/−^ mice), revealed the presence of *Pcyox1* mRNA in atherosclerotic lesions and medial SMCs of Pcyox1^+/+^/Apoe^−/−^ mice, while no staining was evident in Pcyox1^−/−^/Apoe^−/−^ mice (Fig. 3q, r). Co-staining of *Pcyox1* mRNA with cell-type-specific protein markers (F4/80 and alpha-SMA) by dual ISH-IHC demonstrated that both lesional macrophages (Fig. 3s) and SMCs (Fig. 3t) can synthesize PCYOX1.

Regrettably, the rabbit polyclonal antibody HPA035193, which recognizes a portion of the protein encoded by a region of *Pcyox1* gene outside the exon 6, demonstrated immunoreactivity in both Pcyox1^+/+^/Apoe^−/−^ and Pcyox1^−/−^/Apoe^−/−^ mice, likely corresponding to a putative truncated inactive form of the protein^20^. On the other hand, PCYOX1 antibody sc-136391, raised against an AA sequence encoded by exon 6, was not able to detect PCYOX1 protein in mouse sections. Similarly, other commercially available PCYOX1 antibodies did not give reliable IHC results due to either absence of signal or unspecific staining, thus making impossible, at the time of writing, the evaluation of the presence of PCYOX1 protein in the mouse tissues.

### Pcyox1 deficiency retards atheroprogression and reduces lesion vulnerability in Apoe^−/−^ mice

**Fig. 4:**
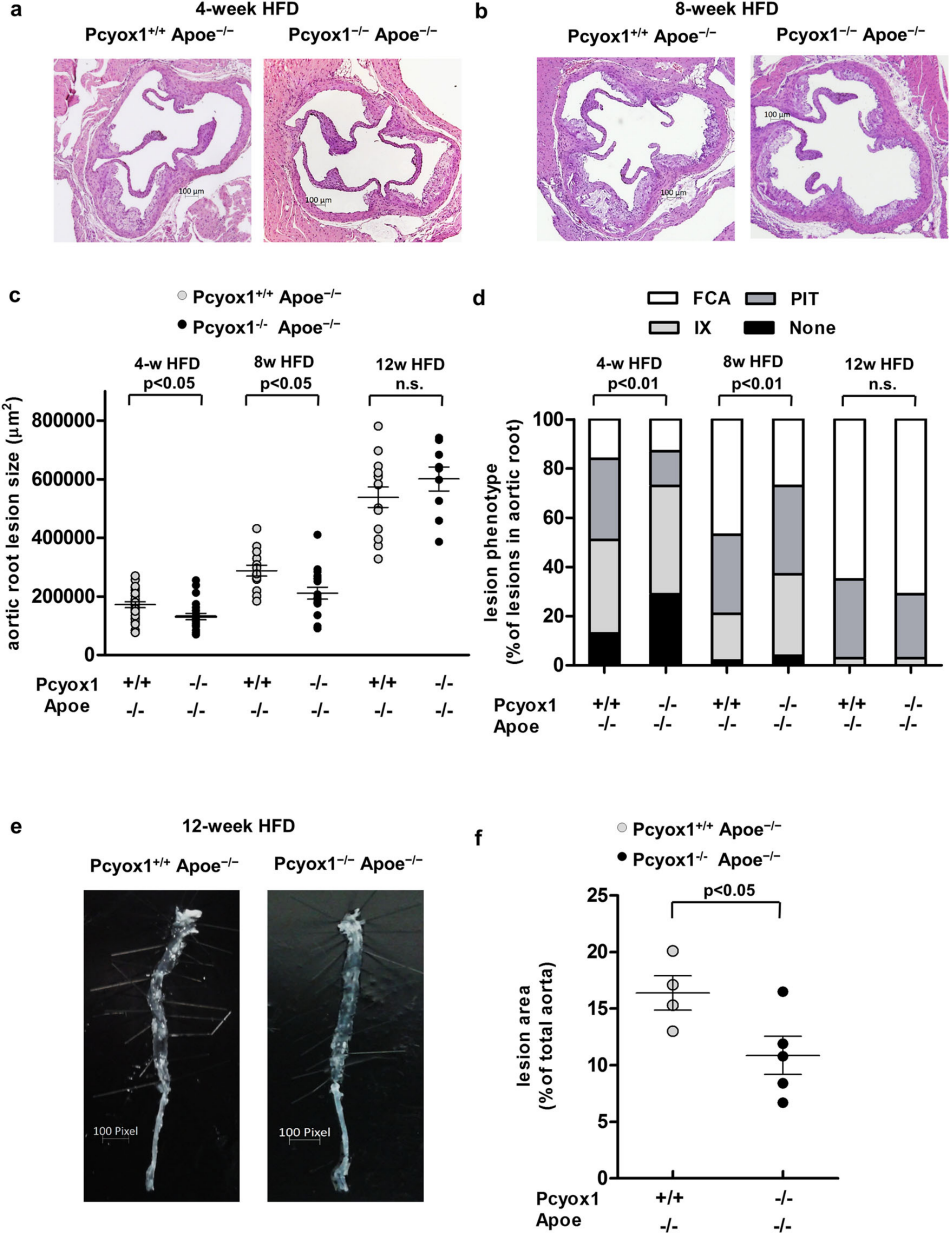
Progression of atherosclerosis is retarded in Pcyox1^−/−^/Apoe^−/−^ mice. Representative images of H&E staining of the aortic root from Pcyox1^+/+^/Apoe^−/−^ and Pcyox1^−/−^/Apoe^−/−^ mice fed a HFD for 4 (**a**) or 8 (**b**) weeks. **c** Quantification of atherosclerotic lesion size. Data are presented as circle plot, with each circle representing an individual mouse and bars showing the mean value ± SEM. **d** Characterization of lesion phenotype according to the atherosclerosis stages: IX intimal xanthoma, PIT pathological intimal thickening, FCA fibrous cap atheroma. Statistical significance was calculated by Fisher exact test. **e**, **f** Representative images and quantification of atherosclerotic areas in the whole aorta by en face analysis. The bright white material is unstained atherosclerotic lesions. Statistical significance calculated by Student’s *t* test. Data are presented as circle plot, with each circle representing an individual mouse and bars showing the mean value ± SEM.

To address the role of Pcyox1 during atherogenesis, we investigated whether Pcyox1 deficiency affected atherosclerotic lesion formation in Apoe^−/−^ mice fed a HFD for 4, 8, or 12 weeks. We found that, in the early and intermediate stages of atherogenesis (4- and 8-week HFD), Pcyox1 deficiency resulted in a significantly smaller lesion area in the aortic root (∼−25% vs respective controls) (Fig. 4a–c). Moreover, Pcyox1 deficiency was associated with less advanced lesion phenotypes compared with controls after 4 and 8 weeks (Fig. 4d), expressed as a percentage of the total number of lesions that appeared as intimal xanthoma—representing early stages—pathological intimal thickening, and fibrous cap atheroma, the latter representing more advanced stages of atherosclerotic lesions^21^. After prolonged HFD feeding (12 weeks), a decrease in plaque area was no longer observed in the aortic root of Pcyox1^−/−^/Apoe^−/−^ mice as compared with controls, and no differences were evident in lesion phenotypes (Fig. 4c, d). In contrast, en face analysis of the whole aorta, which can be more informative in mice with more advanced lesions when it is probably too late a stage to determine differences in lesion formation at aortic sinus^22^, revealed significantly less atherosclerosis burden in Pcyox1-deficient mice (Fig. 4e, f).

**Fig. 5:**
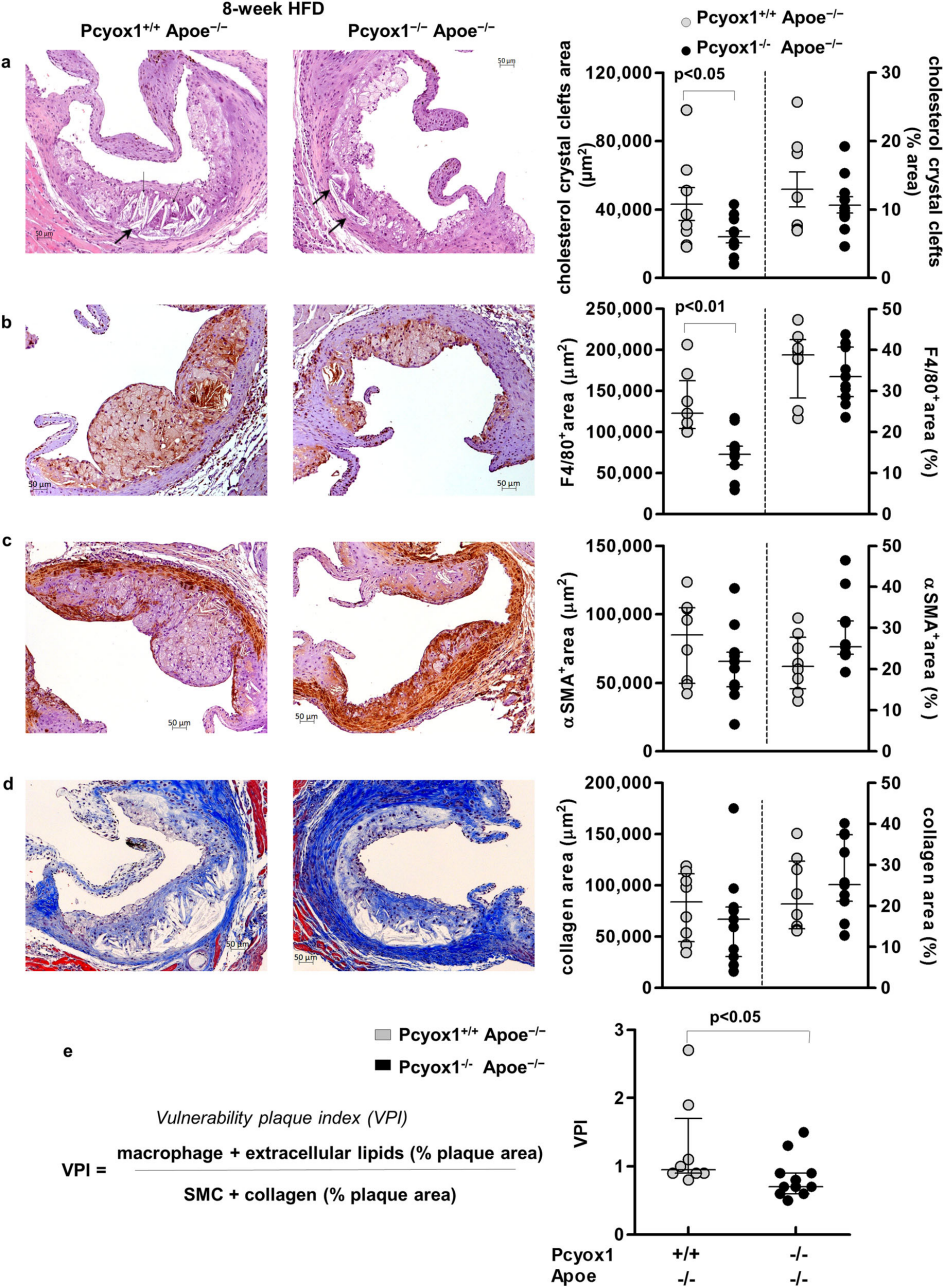
Pcyox1 deficiency is associated with reduced lesion vulnerability. Representative images and quantification of: **a** cholesterol crystal clefts, indicated by arrows (H&E staining); **b** macrophages (F4/80 staining); **c** α-smooth muscle cells (α-SMA staining); **d** collagen (Masson’s Trichrome staining) in atherosclerotic lesions from aortic roots of Pcyox1^+/+^/Apoe^−/−^ (*n* = 8) and Pcyox1^−/−^/Apoe^−/−^ (*n* = 11) mice fed a HFD for 8 weeks. Shown on the right are the results of the morphometric analysis for the different stainings, expressed either as absolute positive areas within the intima or as percentage of plaque that stained for each component. **e** Calculation of vulnerability plaque index. Data are presented as circle plot, with each circle representing an individual sample and bars showing the mean value ± SEM (**a**) or median with interquartile range (**b**–**e**). Statistical significance was calculated by Student’s *t* test (**a**) or Mann–Whitney test (**b**–**e**).

Based on the results of the time-course histological analysis, we next assessed features of lesion vulnerability in aortic root sections of Pcyox1^−/−^/Apoe^−/−^ and Pcyox1^+/+^/Apoe^−/−^ mice after 8 weeks of HFD feeding. Quantification of extracellular lipids (identified as cholesterol crystal clefts) (Fig. 5a), macrophages (Fig. 5b), SMCs (Fig. 5c), and collagen (Fig. 5d) showed that Pcyox1 deficiency favorably modifies the balance between destabilizing components (extracellular lipid and macrophage content) and stabilizing fibromuscular components (SMCs and collagen content), as demonstrated by the integration of these parameters into an overall measure of lesion stability, the vulnerability plaque index (VPI)^23^. Indeed, a significant lower VPI was observed in Pcyox1^−/−^/Apoe^−/−^ compared to Pcyox1^+/+^/Apoe^−/−^ mice (Fig. 5e).

We also found that Pcyox1^−/−^/Apoe^−/−^ mice fed a HFD displayed a significant improvement of plasma lipid profile (Table 1 and Supplementary Fig. 4a–c), a lower body weight (∼14%) observed in the absence of significant differences in food intake (Supplementary Fig. 4d) associated with the reduction of visceral adipose tissue depots (Supplementary Fig. 4e), a lower level of circulating PAI-1 activity, and a lower basal glycemia compared to Pcyox1^+/+^/Apoe^−/−^ mice (−23%) (Table 1). A significant decrease in body weight (∼9%) and a slight decrease of circulating lipids, although not significant, were observed in Pcyox1^−/−^/Apoe^−/−^ mice fed a chow diet in comparison with Pcyox1^+/+^/Apoe^−/−^ (Supplementary Table 3). No significant variation of circulating non-esterified fatty acids, plasma apoB, and any sign of hepatotoxicity, as assessed by alanine transaminase (ALT) and aspartate aminotransferase (AST), were observed (Table 1). Further, by assessing a panel of liver genes relevant to lipid metabolism (see “Methods” section), we found that the expression of peroxisome proliferator-activated receptor γ (*Pparg*) was significantly decreased in the liver of Pcyox1^−/−^/Apoe^−/−^ mice fed a HFD, in comparison to that of Pcyox1^+/+^/Apoe^−/−^ mice (−76%) (Supplementary Fig. 4f).

**Table 1:** Body weight, plasma lipids levels, and other biochemical parameters in Pcyox1^+/+^/Apoe^−/−^ and Pcyox1^−/−^/Apoe^−/−^ fed a HFD for 8 weeks.

Measurement	Pcyox1^+/+^/Apoe^−/−^	Pcyox1^−/−^/Apoe^−/−^	*p* value
Body weight, g	33.6 ± 4.3 (21)	29.0 ± 2.6 (21)	<0.001
Total cholesterol, mg/dL	933 ± 120 (13)	687 ± 191 (18)	<0.001
Non-esterified cholesterol, mg/dL	406 ± 34 (12)	307 ± 84 (18)	<0.001
Cholesterol esters, mg/dL	503 ± 69 (12)	376 ± 112 (17)	<0.01
Triglycerides, mg/dL	218 ± 59 (12)	121 ± 55 (17)	<0.001
Phospholipids, mg/dL	626 ± 77 (13)	481 ± 118 (18)	<0.001
Non-esterified fatty acids, mmol/L	0.256 ± 0.03 (8)	0.195 ± 0.10 (12)	n.s.
Plasma apoB, μg/mL	182 ± 14 (14)	177 ± 28 (18)	n.s.
ALT activity, mU/mL	10.9 ± 2.4 (8)	9.3 ± 1.8 (11)	n.s.
AST activity, mU/mL	14.1 ± 8.6 (8)	13.4 ± 8.2 (12)	n.s.
Glucose, mg/dL	255 ± 36 (13)	197 ± 44 (18)	<0.001
PAI-1, ng/mL	0.371 ± 0.087 (5)	0.194 ± 0.094 (8)	<0.01

Values are represented as mean ± SD. Number of analyzed mice is reported in parenthesis.

### Pcyox1 deficiency is associated with lower levels of inflammation and lipid peroxidation

**Fig. 6:**
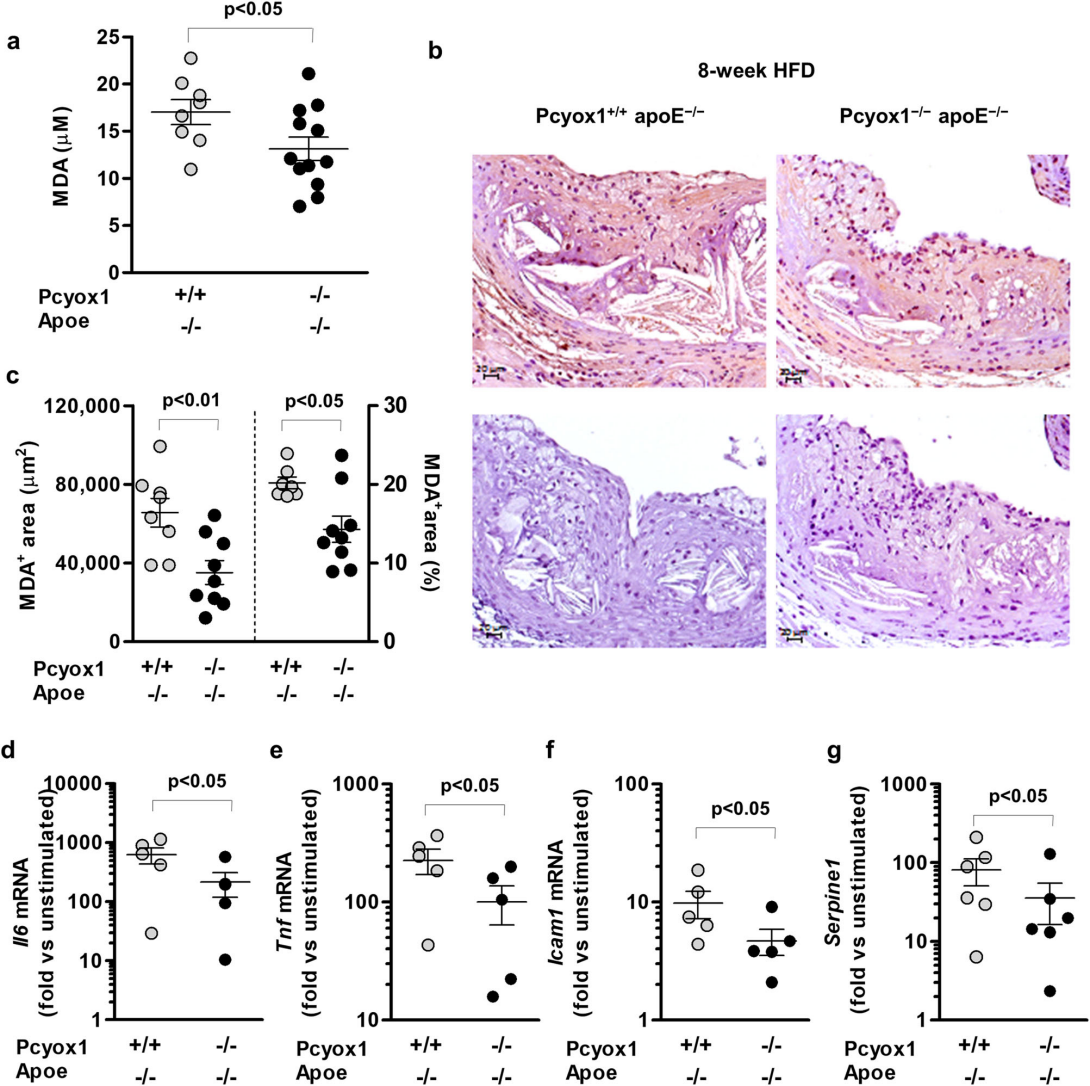
Pcyox1 deficiency is associated with lower levels of lipid peroxidation and decreased LPS-induced inflammatory response in murine peritoneal macrophages. MDA in plasma (**a**) and in the aortic root lesions (**b**, **c**) from Pcyox1^+/+^/Apoe^−/−^ (*n* = 8) and Pcyox1^−/−^/Apoe^−/−^ (*n* = 11) mice fed a HFD for 8 weeks. **b** Representative images of MDA immunostaining (upper panels) and negative control sections incubated with no primary antibody (lower panels); **c** results of the morphometric analysis of MDA-stained sections, expressed either as absolute positive areas within the intima or as the percentage of total lesion area. Data are presented as circle plot, with each circle representing an individual mouse and bars showing the mean value ± SEM. *p* < 0.05 or *p* < 0.01 by Student’s *t* test. **d**–**g** mRNA levels normalized to the housekeeping gene 18S rRNA of *Il6* (**d**), *Tnf* (**e**), *Icam1* (**f**), and *Serpine1* (**g**) in macrophages isolated from Pcyox1^+/+^/Apoe^−/−^ and Pcyox1^−/^^−^/Apoe^−/−^ mice and treated with LPS 5 ng/mL for 2 h. Data are expressed as fold increase induced by LPS with respect to unstimulated cells and presented as circle plot, with each circle representing an individual mouse and bars showing the mean value ± SEM (*n* = 5). *p* < 0.05 by Student’s *t* test.

To further investigate the effect of Pcyox1 deficiency on the characteristics of the atherosclerotic lesion in Apoe^−/−^, we assessed two important features of inflammation: the neutrophil content of the lesions^24,25^^,^ and the expression of NLP family, pyrin domain containing 3 (NLRP3) protein, one of the key components of the NLRP3 inflammasome^26,27^. We found that lesions of Pcyox1-deficient mice showed a minor content of neutrophils (although not statistically significant) and a lower expression of NLRP3 compared to lesions of Pcyox1^+/+^/Apoe^−/−^ (Supplementary Fig. 5), without reduction in the levels of circulating neutrophils and monocytes (Supplementary Fig. 6). As oxidative stress plays a key role in atherogenesis and PCYOX1 is able to generate oxidant species in vitro, we evaluated the effect of Pcyox1 deficiency on lipid peroxidation in vivo by measuring the plasma levels of malondialdehyde (MDA) in mice fed HFD for 8 weeks and the extent of MDA immunopositive area in their aortic root lesions. Pcyox1^−/−^/Apoe^−/−^ mice displayed both lower levels of MDA in plasma (−23% vs control, *p* = 0.05) (Fig. 6a) and less immunostaining for MDA (Fig. 6b, c). In addition, besides the above-described reduced macrophages content in the Pcyox1^−/−^/Apoe^−/−^ atherosclerotic lesion (Fig. 5b), we found that lipopolysaccharide (LPS) stimulation prominently increased the expression of tumor necrosis factor alpha (*Tnf*), *Il6*, intercellular adhesion molecule 1 (*Icam1*), and Serpin family E member 1 (*Serpine1*), coding for PAI-1, in thioglycollate-elicited mouse peritoneal macrophages from Pcyox1^+/+^/Apoe^−/−^ and that the pro-inflammatory impact of LPS was significantly reduced by Pcyox1 deficiency (Fig. 6d–g).

## Discussion

In this study, we provided evidence that PCYOX1 likely represents an emerging player in atherogenesis. We showed that, in the human atherosclerotic lesions, this poorly known lipoprotein-associated protein is highly expressed by vascular cells and accumulates extracellularly in intimal regions rich in apolipoprotein B deposits, as lesion progresses from early to more advanced stages. These findings suggest that PCYOX1 is synthesized locally and mainly transported within the subintimal space by proatherogenic apoB100-containing lipoproteins depositing in the arterial wall, where the enzyme likely reacts with its physiological substrate prenylcysteine (FC) to produce oxidant species. Indeed, vascular cells (endothelial and SMCs), as well as human macrophages do express *PCYOX1* mRNA and cellular protein, while only human hepatocytes are able to secrete it (Supplementary Fig. 7). This pattern of distribution in diseased human vascular tissue suggests that PCYOX1, with its ability to promote apoB100-containing lipoprotein oxidation by pathways involving H_2_O_2_ production, might play a role in the development of the atherosclerotic lesion. This hypothesis is strongly supported by the observation that Pcyox1 deficiency retards atheroprogression in Apoe^−/−^ mice on HFD and is associated with decreased features of lesion vulnerability and lower levels of plasma lipids, and lipid peroxidation, at both systemic and vascular level.

In addition, we provide evidence suggesting that a reduction of inflammation likely contributes to the atheroprotective effect of Pcyox1 deletion. Indeed, the lesions of Pcyox1-deficient mice appear to display less neutrophil content and lower NLRP3 staining compared to lesions of Pcyox1^+/+^/Apoe^−/−^ mice. Further, we found that macrophages of Apoe^−/−^ mice lacking Pcyox1 synthesize less pro-inflammatory cytokines compared to macrophages from control mice. As macrophages contribute to the maintenance of the local inflammatory response by secreting proinflammatory cytokines and chemokines and producing ROS^28^, we can hypothesize that also these cells participate in the PCYOX1-mediated effects. However, our finding is an oversimplification of the complexity of macrophage phenotypes and their functions^29^. Collectively, these findings identify the pro-oxidant enzyme PCYOX1 as an emerging player in atherogenesis and a new candidate target for preventing and treating atherosclerosis-related diseases.

PCYOX1 first emerged at the end of the ‘90s as a lysosomal enzyme involved in the catabolism of prenylated proteins, which represent 2% of all cellular proteins^12^. Afterwards, thanks to the advent of proteomics, the knowledge on PCYOX1 was extended to the finding that this protein belongs to the lipoprotein proteome^10,30^. However, its biological role, besides the catabolism of prenylated proteins, remained largely unknown. A recent systematic review that utilized as sources Pubmed (18 hits found using PCYOX1 and the alternative name prenylcysteine lyase (PCL1), until June 2021) and primarily bioinformatics databases suggests that PCYOX1 might exert multiple roles, which extend beyond the metabolism of prenylated proteins^31^. Nonetheless, apart from the study of Beigneux et al.^20^ showing the absence of any histologic abnormalities in a survey of >30 tissues from Pcyox1-deficient mice on a mixed C57BL/6-129/SvJae genetic background, the in vivo biological roles of PCYOX1 have not been addressed so far.

Our study suggests that PCYOX1 might represent a novel member in the panel of oxidant enzymes involved in the pathogenesis of atherosclerosis. As described by Casey’s group, PCYOX1 is a FAD-dependent thioether oxidase that produces free cysteine, an isoprenoid aldehyde, and a stoichiometric amount of H_2_O_2_^32^. The known ROS-producing systems in the vascular wall currently include nicotinamide-adenine dinucleotide phosphate oxidase, xanthine oxidase, the mitochondrial electron transport chain, and uncoupled endothelial nitric oxide synthase^33^, which can also cross-talk among them, further complicating the scenario (reviewed in ref. ^34^). Unlike these enzymes and other systems responsible for the formation of oxidation-specific epitopes^35^, which are all intracellular or endothelium-bound, as the case of xanthine oxidase^36^, PCYOX1 is, to the best of our knowledge, the only lipoprotein-associated pro-oxidant enzyme with a role in atherogenesis.

The discovery of PCYOX1 as a potential novel oxidant enzyme could help to shed light on the complex nature of oxidative stress generation in the initiation and continuation of the atherosclerotic process. In fact, the extent to which each oxidant enzyme contributes to lipoprotein oxidation in vivo, and thus to atherosclerosis, remains to be fully elucidated, and there is still much we do not understand about the individual processes. Thus, only the discovery of the players in this cross-talk, potential mechanisms, and also the more and more complete understanding of why and how oxidation occurs will help to develop more specific and therefore more powerful antioxidants that may ultimately be more successful than current therapeutic approaches. Indeed, a so-far underestimated reason for the failure of therapies to fight oxidative stress with “classical antioxidants” may be, among others, the complex nature of ROS formation^34^.

The role of PCYOX1 as a regulator of the oxidant system is strengthened by the proteomic analysis of the PCYOX1 silenced-cells, in which the most relevant modulated processes were represented by the response to oxidative stress and the response to ROS. Moreover, additional information emerging from the secretome analysis revealed that PCYOX1 is a multifunctional protein potentially involved in a plethora of systems, including the regulation of peptidase activity, platelet degranulation, regulation of signal transduction, response to stress, regulation of response to stimulus, inflammatory response, and response to wounding. However, further investigations are needed to verify whether and understand how PCYOX1 can modulate such different processes, which might not be restricted to its pro-oxidant activity only. Insights into the functions of PCYOX1 can be gained by systematic analyses of large-scale protein–protein interaction networks. For example, PCYOX1 may potentially interact with other proteins through multiple domains, thereby potentially coordinating diverse biological functions (https://thebiogrid.org/119547/summary/homo-sapiens/pcyox1.html).

As discussed before, besides its unique catalytic mechanism, another key feature of PCYOX1 is its association with lipoproteins, including VLDL, LDL, and, to a lesser extent, HDL. In this study, we provide evidence that PCYOX1 likely binds to nascent lipoproteins in the ER, and once bound, it can produce H_2_O_2_, which in turn oxidizes the apoB100-containing lipoprotein itself. The finding that PCYOX1 is more abundant in VLDL than LDL deserves some considerations. Of interest, VLDL-associated apolipoproteins and predominant lipids emerged as the strongest determinants of CVD risk^37^. Further, recent data indicate that all apoB-containing lipoprotein particles, including VLDL particles and their metabolic remnants, as well as LDL particles, have approximately the same effect on the risk of CVD per particle^38^. The mechanisms underlying the atherogenic effects of VLDL have been poorly investigated; interestingly, in vitro experiments have demonstrated that VLDL contributes more strongly to nuclear factor-κB activation than LDL or extensively oxidized lipoproteins^39^. Thus, the differential distribution of PCYOX1 in various plasma lipoproteins may have important functional implications, and much effort should be spent elucidating the molecular basis for these associations. The association of the enzyme with lipoproteins may be a major determinant of its catalytic efficiency in vivo. For example, it is possible that the lipoprotein lipids facilitate the access of hydrophobic substrates to the active site of the enzyme, which may account for the efficiency of substrate hydrolysis when the enzyme is bound to lipoproteins. In addition, the association of PCYOX1 with lipoproteins could allow this enzyme to circulate in the blood and gain unfettered access to the vascular wall. Herein, PCYOX1 interacts with the physiological substrate, prenylcysteine, which derives from the catabolism of proteins affected by prenylation, an ubiquitous, stable, irreversible covalent posttranslational modification that regulates membrane interactions and biological activities of a variety of cellular proteins^12,40^. Based on these assumptions, preventing PCYOX1 binding to lipoproteins could represent a strategy to block its physiological function. Anyway, its preferential association with atherogenic apoB100-containing lipoproteins supports the proatherogenic role of PCYOX1. Therefore, identifying the factors that define PCYOX1’s association with lipoproteins is an important issue with clear-cut physiologic consequences.

We also showed that Pcyox1-deficient mice exhibited a reduced weight gain associated with a lower accumulation of visceral adipose tissue depot. Further investigations might reveal whether PCYOX1 has a role in adipogenesis and/or energy expenditure that could justify the reduction in the visceral fat depot and whether the visceral fat is a mediator of the observed metabolic changes in Pcyox1-deficient mice. The visceral adipose tissue is indeed considered the most diabetogenic and atherogenic fat depot^41^, and it has been demonstrated that this tissue accelerates atherosclerosis in apolipoprotein E-deficient mice^42^. The visceral fat is indeed characterized by chronic low-grade inflammation and secretion of inflammatory cytokines and chemokines, which represent a potent stimulus for promoting vascular complications^41,42^.

In this regard, we observed in Pcyox1^−/−^/Apoe^−/−^ mice a significant decrease in plasma activity of PAI-1, the primary physiological inhibitor of fibrinolysis^43,44^. It has been suggested that the adipose tissue is a primary source of PAI-1 since its plasma levels correlated with the amounts of visceral fat and serum triglycerides^45,46^ and, vice versa, the weight loss is followed by an improvement in fibrinolytic activity due to a decrease in PAI-1 levels^47,48^. Further, in vitro assays revealed an impaired adhesion of human platelets to both fibrinogen-coated plates and to human endothelial cells mediated by factors, among which is PAI-1, present in the secretome of PCYOX1-silenced cells. Altogether these data pave the way for future studies aimed at investigating the role of PCYOX1 in atherothrombosis. The observations that PAI-1 expression is lower in macrophage-derived from Pcyox1^−/−^/Apoe^−/−^ mice broadens the bio-pathophysiological functions of the axis PCYOX1–PAI-1 to fibrosis, cell regeneration, and inflammation, and metabolic or neurological disorders^49–51^.

It also remains to be elucidated the mechanism(s) underlying the improvement in the glucose levels observed in Pcyox1^−/−^/Apoe^−/−^ mice. Whether the pancreatic islet inflammation^52^, the defects in insulin secretion rather than defects in insulin resistance, play a role in the metabolic disturbs that are attenuated by Pcyox1 deficiency will be addressed in future studies. Mechanistically, it remains to be addressed whether reduced atherosclerosis associated with Pcyox1 deficiency could be the result of systemic changes in adipose tissue and lipid–glucose metabolism. In this regard, the lower lipid levels in the double knockout mice may be sufficient to explain the decrease in atherosclerosis development. Indeed, despite plausible effects on oxidation, improved dyslipidemia may be a sufficient reason and question the postulation that PCYOX1 is a novel therapeutic target. In addition, the finding that, among different genes involved in lipid metabolism, *Pparg*, which is upregulated in the liver under conditions of nutrient overload and obesity^53–55^, is significantly decreased in the liver of Pcyox1-deficient mice suggests an additional contribution of the hepatic lipid homeostasis.

Finally, since PCYOX1 was not reduced in vitro by both fibrate and statin treatment (Supplementary Fig. 8), our results indicate that PCYOX1 might represent an additional drug target in atherosclerosis. We could add it to the list of the recently discovered new targets with mechanistic links to atherothrombotic heart disease, such as proteins involved in glycoprotein recognition and clearance, regulators of triglyceride-rich particle metabolism, inflammatory pathways that impair efferocytosis, and the gut microbiome^56^. Surely, established lipid-lowering therapies, predominantly statins, have delivered tremendous clinical and societal value by lowering LDL cholesterol, thereby preventing atherosclerotic CVD and mortality. However, currently available lipid-lowering therapies are insufficient to bring a halt to the CVD epidemic for several reasons: first, side effects precluding a substantial proportion of individuals from receiving adequate doses; second, scarce adherence to statin therapy; third, limited efficacy of statins (even high-intensity statin therapy in combination with ezetimibe would leave up to a sixth of patients in need of additional lipid lowering). Finally, although a new effective option has recently become available in the form of therapeutic mAb inhibitors of proprotein convertase subtilisin/kexin type 9 (PCSK9), most patients who need additional lipid-lowering are unable to receive PCSK9-inhibiting mAbs because of high cost and relevant injection burden^57^. Thus, there is still a significant unmet need for effective and accessible anti-atherosclerotic therapies.

In conclusion, PCYOX1 has biological functions that extend beyond its fundamental role in the prenylation process, with contributions to inflammation, thrombosis, and atherosclerosis. Understanding the biology and mechanisms of all functions of this unique enzyme is likely to provide additional therapeutic opportunities in addressing atherosclerosis.

## Methods

### Reagents

All reagents and chemicals were purchased from Sigma Aldrich, Milan, Italy, if not expressly declared.

### Cell cultures

The human hepatoma cell line, HepG2, were cultured as previously described^58^. HAECs were purchased from Cambrex and cultured in Endothelial Cell Growth Medium 2 (EGM-2) according to the supplier’s instructions. The Flp-In™-CHO cell line, containing a single integrated FRT site and stably expressing the lacZ-Zeocin™ fusion gene from the pFRT/lacZeo2 plasmid, were cultured according to the supplier’s instructions (Flp-In™-CHO R758-07, Life Technologies, ThermoFisher Scientific). Human primary hepatocytes (Lonza) were cultured in Biocoat Collagen I multiwell plates (Corning) using the maintenance medium (HBM) according to the supplier’s instructions. Human aortic SMCs (Lonza) were cultured in complete SMBM2 medium (Lonza) according to the supplier’s instructions. Human umbilical vein endothelial cells (HUVECs) were purchased from Lonza and cultured in EGM-2 according to the supplier’s instructions.

### Culture of monocyte-derived macrophages (MDMs)

MDMs were obtained from differentiation of monocytes isolated from peripheral blood of healthy consenting subjects as described^59^. Briefly, mononuclear cells were isolated by on Ficoll-Paque (GE Healthcare, EuroClone) density centrifugation at 540 × *g* for 20 min at room temperature and plated (2 × 10^6^/mL) in 35 mm well plates (Primaria^TM^, Falcon, Sacco S.r.l). After 90 min, non-adherent cells were removed, and adherent monocytes were cultured over 7 days in Medium 199 (Lonza, EuroClone, Milan, Italy) supplemented with 2 mmol/L L-glutamine, 100 U/mL penicillin, and 100 μg/mL streptomycin and containing 10% autologous serum freshly obtained from blood clotted for 2 h at 37 °C. The medium was not replaced throughout the culture period.

### Stable transfection of PCYOX1 short hairpin RNA (shRNA) in HepG2 cells

Stable gene silencing of PCYOX1 was obtained in HepG2 cells using shRNA Plasmids (Santa Cruz Biotechnology), a pool of 3 target-specific lentiviral vector plasmids each encoding 19–25 nt (plus hairpin) shRNAs designed to knock down PCYOX1 gene expression. A shRNA plasmid encoding for a scrambled shRNA sequence that does not lead to the specific degradation of any cellular message was used as negative control (control cells). Cells were transfected at 50–70% confluency for 24 h with 0.5 µg of plasmid and 2 µL of transfection reagent (Santa Cruz Biotechnology) for each well of a 6 well/plate and then grown in complete medium containing 1 µg/mL puromycin to enable selection.

### PCYOX1 overexpression in CHO cells

An empty pcDNA5/FRT vector and a custom pcDNA5/FRT:PCYOX1 (Invitrogen, Life Technologies) were transfected into Flp-In-CHO cells (R758-07, Invitrogen, Life Technologies) with a pOG44 expression vector (V6005-20, Invitrogen, Life Technologies) and the PCYOX1-expressing clones were selected according to the Flip-In System protocol (K6010-01, Invitrogen, Life Technologies). All experiments were performed using cells within 20 passages from transfection and selection, comparing PCYOX1-overexpressing cells with CHO cells transfected with empty vector, named control cells.

### Immunocytochemistry

In order to investigate the subcellular localization of PCYOX1, we performed double immunofluorescence staining of HepG2 cells with a mouse mAb raised against amino acids 356–478 of PCYOX1 of human origin (sc-136391, 1:200 in Dako antibody diluent; Santa Cruz Biotechnology) and a panel of validated mouse mAbs against organelle markers (Prestige Antibodies Organelle Markers Sigma). The panel included the following antibodies: anti-NOP56 (AMab91013, 1:500 in Dako antibody diluent, a marker for nucleoli); anti-GORASP2 (AMAb91016, 1:500 in Dako antibody diluent, a marker for Golgi apparatus); anti-PDIA3 (AMAb90988, 1:500 in Dako antibody diluent, a marker for ER); anti-TUFM (AMAb90964, 3 μg/mL, a marker for mitochondria). In addition, co-staining of PCYOX1 and lysosomes was performed using a rabbit polyclonal anti-LAMP1 antibody, Cy3 conjugate (1:1000 in Dako antibody diluent, Sigma, Cat. No. L0419). Cells grown on glass coverslips were fixed in 4% paraformaldehyde in phosphate-buffered saline (PBS) for 20 min at room temperature, washed with PBS, and subjected to heat-mediated antigen retrieval (HIER) using Dako Target Retrieval citrate buffer, pH 6.0, prior to commencing immunofluorescence procedure. After rinsing in PBS, cells were permeabilized with 0.3% Triton X-100 in PBS for 15 min at room temperature, and incubated with 3% bovine serum albumin (BSA) and 20 mg/mL glycine in PBS for 60 min at room temperature to block non-specific antibody binding. Cells were then incubated in the PCYOX1 antibody (1:200 dilution in Dako antibody diluent) in a humidified chamber overnight at 4 °C. After being rinsed three times for 10 min each in PBST (PBS with 0.1% Tween 20), coverslips were incubated with an Alexa Fluor 488 donkey anti-mouse IgG secondary antibody (1:200 dilution in Dako antibody diluent) at room temperature for 90 min and were then thoroughly rinsed in PBST. Coverslips were then incubated again with 3% BSA and 20 mg/mL glycine in PBS for 60 min, followed by incubation with Rodent Block M (Biocare Medical) for 45 min to block for mouse IgG. Coverslips were then washed in PBST and incubated with the different Prestige Antibodies Organelle Markers (2 µg/mL in Dako antibody diluent) at room temperature for 60 min, followed by detection with an Alexa Fluor 546 donkey anti-mouse IgG secondary antibody (1:500 dilution in Dako antibody diluent) at room temperature for 90 min. For double immunofluorescence with the rabbit polyclonal anti-LAMP1 antibody, Cy3 conjugate, the incubation with Rodent Block M was omitted. After immunostaining for PCYOX1, coverslips were blocked with 3% BSA and 20 mg/mL glycine, and coverslips were detected with the antibody (1:1000 dilution in Dako antibody diluent) at room temperature for 60 min. At the end of the immunostaining protocol, coverslips were washed and mounted with VECTASHIELD® Antifade Mounting Medium with 4,6-diamidino-2-phenylindole (DAPI; Vector Laboratories). Fluorescence images were taken under an Axiovert Observer.1 (Zeiss). Optical sections were generated using the ApoTome device and photographed with an Axiocam MRm camera (Zeiss). Appropriate control coverslip sections to check for unspecific staining were included in each experiment. Specifically, coverslips have been treated: only with secondary antibodies, omitting from the sequence of incubation both the primary antibodies, to exclude that secondary antibodies bind to tissue; omitting from the sequence of incubation the first secondary antibody and the organelle-specific primary antibody, to exclude that secondary antibody binds to the inappropriate primary antibody; omitting from the sequence of incubation the organelle-specific primary antibody, to exclude that secondary antibody binds to inappropriate primary antibody or secondary antibodies bind to each other.

### Cell proliferation assay and apoptosis

Cell proliferation was evaluated using the cell proliferation enzyme-linked immunosorbent assay (ELISA), bromodeoxyuridine (Roche Diagnostics), in accordance with the manufacturer’s instructions. The 3-[4,5-dimethylthiazol-2-yl]-2,5 diphenyl tetrazolium bromide assay was based on the protocol first described by Mosmann^60^. Apoptosis was detected by quantification of histone-complexed DNA fragments (mononucleosomes and oligonucleosomes) using a one-step sandwich immunoassay (Roche Diagnostics), according to the manufacturer’s instructions.

### Intracellular ROS formation

HepG2 cells and CHO cells cultured on 96-well plates were loaded with 10 mmol/L 29,79-dichlorofluorescein diacetate (DCFH-DA) for 1 h at 37 °C. After incubation, cells were washed in PBS and left in serum-free phenol-free medium for the indicated times, as previously described^61^. ROS production was measured at different time points (from 0 to 180 min) on the basis of the intensity of DCF emission at 535 nm (excitation 485 nm) in microplate reader Infinite 200 (TECAN). The results are expressed as the percentage increase in fluorescence after 180 min in comparison with the basal fluorescence (T0) of each sample after blank subtraction (wells with cells without DCF).

### Lipoprotein isolation and treatment

Lipoprotein were isolated from plasma or cell supernatant by density gradient ultracentrifugation as described^10^. In particular, lipoproteins (*d* < 1.063) were isolated from cell conditioned media of control or PCYOX1-silenced HepG2 cells. Protein concentrations were determined with the DC Protein Assay Kit (Bio-Rad Laboratories). In order to evaluate the effects of PCYOX1 activity, VLDL or LDL (100 µg/mL) were incubated with FC (200 µmol/L)^10^ and FeCl_2_ (100 μmol/L)^62^, or vehicle (plus FeCl_2_), for 72 h.

### Uptake of FC-LDL by MDMs

FC-treated LDL (0.2 mg/mL protein) was mixed with equal volume of lipoprotein-deficient serum (LPDS, 0.5 mL) and then filter (0.22 µm) sterilized. A 50-µL aliquot of DiO:3,3′-dioctadecyloxacarbocyanine perchlorate (DiO) (Invitrogen, Carlsbad, CA) at 3.0 mg/mL in dimethyl sulfoxide (Sigma, St. Louis, MO) was added to the FC-treated LDL-LPDS mixture. The mixture was then gently mixed and incubated at 37 °C for 16 h. To isolate the labeled FC-LDL-DiO, the density of the solution containing the fluorescent-labeled LDL was raised to 1.21 g/mL with solid KBr followed by density ultracentrifugation gradient and dialyzed against several changes of PBS. MDMs were incubated with FC-LDL-DiO and vehicle-LDL-DiO (both 50 μg/mL protein) for 24 h monitoring green fluorescence, phase contrast with the IncuCyte instrument (Sartorious), every 20 min for a total of 24 h, in order to evaluate the lipoprotein uptake and cell size.

### PCOYX1 activity assay

H_2_O_2_ produced in the PCYOX1 reaction was measured as previously described^10^ using the Amplex Red Kit (Life Technologies). In order to perform the assay, lipoprotein fraction samples were extensively dialyzed against reaction buffer in glycerol-free Slide-A-Lyzer™ Dialysis Cassettes with a 3.5 KDa cut-off for 24 h. Cells were lysed in a hypotonic buffer (10 mmol/L Tris pH 7.3, 10 mmol/L KCl, 1.5 mmol/L MgCl_2_), sonicated, and centrifuged at 9000 × *g* for 30 s. Lipoprotein (15 µg) or cell lysates (25 µg) were incubated at 23 °C in the reaction buffer, in the presence of the PCYOX1 substrate FC (125 µmol/L). The conversion of Amplex Red by H_2_O_2_ into the oxidation product resorufin was measured following the fluorescence on a microplate reader Infinite 200 (TECAN), equipped for excitation in the range of 530 ± 12.5 nm and fluorescence emission detection at 590 ± 17.5 nm. Results are calculated as picomoles of H_2_O_2_ per microgram of proteins produced in the presence of FC. For each point of the standard curve, the value derived from the no-H_2_O_2_ control was subtracted to create a linear regression curve. Blank samples, run with each sample in the absence of FC, were subtracted at all time points. H_2_O_2_ production from PCYOX1 in biological samples was calculated extrapolating from the standard curve the difference in fluorescence units between the samples treated with FC and the one incubated with ethanol (vehicle).

### TF activity

TF activity was measured on cell lysates with the Actichrome TF Activity Kit (American Diagnostica, Stamford, CT, USA) as described^63^.

### Agarose gel

VLDLs incubated with FC (200 µmol/L) were then subjected to electrophoresis on an agarose gel as described^17^. Briefly, the electrophoretic mobility of lipoproteins was determined on agarose gels (1%) in Tris 0.5 mol/L (pH 8.6). Sample loading was 10 µg lipoprotein per lane, and electrophoresis was carried out for 90 min at 100 V. Visualization was done by staining with Sudan Black B (0.1% w/v) in 70% ethanol.

### Real-time quantitative reverse transcriptase PCR (qRT-PCR)

Total cellular and tissue RNA were extracted with the Total RNA Purification Kit (Norgen Biotek Corp.) and reverse transcribed (1 μg) as previously described^63^. Tissues were homogenized by means of TissueLyser II (QIAGEN) before RNA isolation. The quality of RNA was checked by the Agilent 2100 Bioanalyzer system. Amplification of 18S ribosomal RNA was used to correct for fluctuations in input RNA levels and the efficiency of the reactions. Real-time qRT-PCR was performed in triplicate with 2.5 μL of cDNA incubated in 22.5 μL IQ Supermix containing primers and SYBRGreen fluorescence dye (Bio-Rad Laboratories, Milan, Italy) using the iCycler Optical System (Bio-Rad Laboratories, Milan, Italy). The sequences of primers used as normalizer were: human 18S forward: 5′-CGG CTA CCA CAT CCA AGG AA-3′; human 18S reverse: 5′-CCT GTA TTG TTA TTT TTC GTC ACT ACC T-3′; mouse 18s forward: 5′-GTA ACC CGT TGA ACC CCA TT-3′; mouse 18s reverse: 5′-CCA TCC AAT CGG TAG TAG CG-3;′Mouse *Pparg* forward: 5’-TCG CTG ATG CAC TGC CTA TG-3’, Mouse *Pparg* reverse: 5’-GAG AGG TCC ACA GAG CTG ATT-3’; Mouse 3′-hydroxylmethyl glutaryl coenzyme A reductase (*Hmgcr*) forward: 5’- TGT TCA CCG GCA ACA ACA AGA-3’, Mouse *Hmgcr* reverse: 5’-CCG CGT TAT CGT CAG GAT GA-3’; Mouse liver X receptor alpha (*LXRα*) forward: 5’- CTC AAT GCC TGA TGT TTC TCC T-3’; Mouse liver X receptor alpha *(LXRα*) reverse: 5’- TCC AAC CCT ATC CCT AAA GCA A-3’; Mouse LDL receptor (*Ldlr*) forward: 5’- TGA CTC AGA CGA ACA AGG CTG-3’; Mouse *Ldlr* reverse: 5’- ATC TAG GCA ATC TCG GTC TCC-3’; Mouse stearoyl CoA desaturase 1 (*Scd1*) forward: 5’- TTCTTGCGATACACTCTGGTGC-3’; Mouse *Scd1* reverse: 5’- CGG GAT TGA ATG TTC TTG TCG T-3’; Mouse sterol regulatory element binding protein 2 (*Srebf-2*) forward: 5’- TGG GCG ATG AGC TGA CTC T-3’; Mouse *Srebf-2* reverse: 5’- ACT GTA GCA TCT CGT CGA TGT-3’; Mouse lipoprotein lipase (*Lpl*) forward: 5’- TTG CCC TAA GGA CCC CTG AA-3’; Mouse *Lpl* reverse: 5’- ACA GAG TCT GCT AAT CCA GGA AT-3’; Mouse *Cd36* forward: 5’- ATG GGC TGT GAT CGG AAC TG-3’; Mouse *Cd36* reverse: 5’- AGC CAG GAC TGC ACC AAT AAC-3’; Mouse *Gapdh* forward: 5’- CGT GCC GCC TGG AGA AAC C-3’; Mouse *Gapdh* reverse: 5’- TGG AAG AGT GGG AGT TGC TGT TG-3’. Other specific primers were purchased from QIAGEN: Human *PCYOX1*−1, QT 0032228; Human *LDLR*, QT00045864; Mouse *Il6*, QT00098875; Mouse *Tnf*, QT00104006; Mouse acetyl-Coenzyme A carboxylase alpha, QT00258419; Mouse Fatty acid synthase, QT 00149240. Expression levels were calculated by Ct values normalized to the housekeeping gene 18S rRNA or *GAPDH* using the 2^−ΔΔCT^ data analysis method. For visualization of *PCYOX1* amplicons in the different primary cells, PCR products were separated by gel electrophoresis in agarose gel 2% w/v containing GelRed (Biotium) and visualized with Gel doc (Bio-Rad Laboratories).

### Label-free mass spectrometry (LC-MS^E^) analysis

Proteomic analysis of secretome samples was performed after desalting, concentration, and digestion as previously described^64^ with minor variations. Briefly, the cell culture media from each condition were collected and cell debris was removed by centrifugation. Then samples were dialyzed at 4 °C using a 3500 Da molecular weight cut-off dialysis tubing (Spectrum Laboratories) against 5 mmol/L NH_4_HCO_3_ containing 0.01% EDTA, followed by dialysis against water. After lyophilization, the secreted protein pellets were dissolved in 25 mmol/L NH_4_HCO_3_ containing 0.1% RapiGest (Waters Corporation), sonicated, and centrifuged at 13,000 × *g* for 10 min. Samples (50 μg of protein) were then incubated 15 min at 80 °C and reduced with 5 mmol/L dithiothreitol at 60 °C for 15 min, followed by carbamidomethylation with 10 mmol/L iodoacetamide for 30 min at room temperature in the darkness. Then 1 μg of sequencing-grade trypsin (Promega) was added to each sample and incubated overnight at 37 °C. After digestion, 2% TFA was added to hydrolyze RapiGest and inactivate trypsin. Tryptic peptides were used for label-free mass spectrometry analysis, LC-MS^E^, performed on a hybrid quadrupole-time of flight mass spectrometer coupled with a nanoUPLC system and equipped with a Trizaic source (Waters Corporation) as previously detailed^64,65^. Statistical analysis has been performed by means of Progenesis QIP v 4.1 (Nonlinear Dynamics)

### GO analysis

Proteomics data were analyzed with the Search Tool for the Retrieval of Interacting Genes/Proteins (STRING 10.5) database^66^ as previously described^67^ to identify enriched GO terms in the biological process, molecular function, or cellular component categories. In particular, we employed the enrichment function of STRING that calculates an enrichment *p* value based on hypergeometric test using the method of Benjamini and Hochberg for correction of multiple testing (*p* value cut-off of <0.05).

### Platelet adhesion assay

Platelets were isolated from healthy volunteers as previously described^68^. In brief, fresh blood was drawn from an antecubital vein using a 19-gauge needle without venous stasis and collected into Vacutainer tubes containing acid–citrate–dextrose 15% v/v (trisodium citrate 22.0 g/L; citric acid 8.0 g/L; dextrose 24.5 g/L) as anticoagulant. The blood was centrifuged for 10 min at 130 × *g* at room temperature in order to obtain platelet-rich plasma (PRP). The upper two-third of the PRP were recovered in a new tube in the presence of prostaglandin PGE1 (4 µM) and EDTA (10 mmol/L) to allow minimal activation. Platelets were then stained with calcein acetoxymethyl ester (calcein-AM, 2 μg/mL, Life Technologies) for 1 h at 30 °C in the dark. Following the removal of the plasma by centrifugation at 1000 × *g* for 10 min at room temperature, the platelets were washed in Tyrodes-HEPES (140 mmol/L NaCl, 2.6 mmol/L KCl, 1 mmol/L MgCl_2_, 12 mmol/L NaHCO_3_, 0.4 mmol/L Na_2_HPO_4_, 20 mmol/L HEPES, and 0.1% glucose, pH 7.4) plus PGE1 (1 µmol/L) and EDTA (5 mmol/L), centrifuged at 1000 × *g*, and resuspended in Tyrodes-HEPES in the absence of PGE1 but in the presence of Apyrase (0.02 U/mL).

Platelet adhesion onto fibrinogen (FBG)-coated plates^69^ was tested in the presence of conditioned medium from control and PYCOX1 silenced HepG2 cells. Briefly, 96-well black plates were coated for 16 h at 4 °C with 100 μg/mL FBG (dissolved in phosphate buffer 0.04 mol/L containing 0.15 mol/L NaCl) and then blocked with 5% BSA. Calcein-stained platelets (9 × 10^6^) were dissolved in Tyrodes-HEPES buffer with apyrase and incubated for 30 min at 37 °C before addition to the FBG-coated plate containing conditioned media for 30 min at 37 °C. After 3 washes with PBS, bound platelets were lysed with 85 μL of lysis buffer (150 mmol/L NaCl, 10 mmol/L Tris-HCl, pH 7.5, 5 mmol/L EDTA, 1% (w/v) Nonidet P-40) and quantified in a fluorescent plate reader (excitation filter 492 nm, emission filter 535 nm, TECAN Italia, Cernusco SN, Milano, Italy).

Platelet adhesion to endothelial cells was tested seeding 2 × 10^4^ HUVECs in a 96-well black plate and grown until confluent. After 16 h of incubation with conditioned medium from control and PYCOX1 silenced HepG2 cells, washed platelets (6 × 10^6^) preloaded with calcein-AM were added, and allowed to adhere to the cells for 40 min at 37 °C. As described above, fluorescence was measured.

### Immunoblotting

For immunoblotting protein extract, dissolved in Laemmli buffer containing the Protease cocktail inhibitor (Sigma), were electrophoretically separated on a 12% sodium dodecyl sulfate-polyacrylamide gel, transferred to nitrocellulose membranes, and processed as previously described^58^. After transfer to nitrocellulose membrane, total proteins were stained with the MEMcode Protein Staining Kit (Thermo Scientific, Milan, Italy) according to the manufacturer’s instructions. As primary antibodies, we used: mouse anti-PCYOX1(25) (1:500, Santa Cruz Biotechnology); rabbit anti apoB100 (R&D Systems, 1:500 in Tris-buffered saline with Tween 20 0.1% (TBST) with 5% skim milk); mouse anti-HNE (R&D Systems, 1:500 in TBST with 5% skim milk). As secondary antibodies, we used: goat anti-rabbit horseradish peroxidase (HRP) conjugated (Bio-Rad Laboratories) or goat anti-mouse HRP conjugated (Sigma Aldrich). The bands were visualized by means of enhanced chemiluminescence (GE Healthcare) and analyzed with the QuantityOne software (Bio-Rad Laboratories) for densitometric analysis including normalization for total protein loading visualized with MEMcode^70^.

### Immunoenzymatic assays

The concentration of human, GDF-15, THSP1, and CXCL8 was measured using specific immunoassays from R&D system (Biotechne). Human PAI immunoassay was from BIOMEDICA Diagnostics. Mouse PAI-1 activity using ELISA kit (Molecular Innovations) was measured according to the manufacturer’s instructions on citrate plasma. Protein carbonyl measurements were performed by Zentech PC test ELISA (Biocell).

### Collection of human arterial tissues

Samples from subjects with atheromatous plaques (*n* = 15; 10 males, mean age 54.5 ± 7.8 years; 5 females, mean age 57.4 ± 8.6 years) or without evidence of atherosclerosis (*n* = 4, 3 males, mean age 44.7 ± 17.1 years and 1 female 46 years) were harvested from the ascending aorta, the aortic arch, or the innominate artery of donors with normal echocardiographic parameters, excluded from transplantation for technical reasons during multiorgan explantation (cold ischemia time of 4–12 h) at the Cardiovascular TissueBank, Regione Lombardia, of Monzino Cardiologic Center (Milan, Italy). The study protocol was approved by the Ethics Committee of Monzino Cardiologic Center and was conducted in accordance with the principles laid down in the Declaration of Helsinki. All samples were harvested after that organ transplantation consensus was obtained from the donors’ relatives. Tissue samples were immersion-fixed overnight in 10% neutral buffered formalin, embedded in paraffin, and cut transversally into 4-μm sections. When necessary, samples were decalcified using the Bone Decalcification Buffer (ACD catalog 321918) according to the manufacturer’s instructions.

### Mice and diets

Pcyox1^−/−^ mice (kindly provided by Professor Stephen G. Young^20^) were backcrossed four times into a C57/BL6J background mice by using the Marker-Assisted Accelerated Backcrossing (MAX-BAX®) technique (Charles River Laboratories) and subsequently bred with apoE^−/−^ mice (B6.129P2-Apoetm1Unc/J, stock 002052, JAX™ Mice Strain). Intercrosses of resulting Apoe^−/−^/Pcyox1^+/−^ mice yielded offspring that entered the study. All procedures were approved by the Institutional Animal Care and Ethics Committee of the University of Milan and of the Ministry of Health DGSAF (N. 394/2015-PR).

Mice were housed in an air-conditioned room at 22 °C ± 0.5 °C with a 12-h lighting cycle and a free access to food and water. For experiments, double knockout mice (Pcyox1^−/−^/Apoe^−/−^) and control mice (Pcyox1^+/+^/Apoe^−/−^) were fed ad libitum with a HFD containing 0.2% cholesterol, 21.2% fat (42% kcal), and 17.5% protein by weight, (Teklad diet TD.88137; Envigo) beginning at 10 weeks of age for 4, 8, or 12 weeks before atherosclerosis was assessed or for 8 weeks with chow diet. After the indicated time period on HFD, mice were anesthetized by intraperitoneal injection of ketamine hydrochloride (75 mg/kg) and medetomidine (1 mg/kg). The arterial tree was perfused in situ with saline and organs were harvested. Food intake was monitored daily in individual cages by manual weighting of food pellets.

Animals (littermates) entered the experimental procedure when reaching 10 weeks of age. Each experimental session included animals of both genotypes. Results were consistent through the different experimental sessions. Randomization is not relevant in our animal study since the allocation of animals in experimental groups were made based on mouse genotype. Investigators were blinded to group allocation during all the stages of the conduct of the experiments and outcome measure assessment.

### Pcyox1 genotyping

Mice were genotyped for Pcyox1 deficiency by PCR of genomic DNA. For both the wild-type and the knockout alleles, we used the following common forward primer: *Pcyox1*-com, CAGGACCTCTGGAAGAGCAGTCAG. The reverse primers for the wild-type and the knockout alleles, respectively, were as follows: *Pcyox1*-wt, GATCCTTGGGTCTGGAGCTGAAG; and *Pcyox1*-ko, GGGTTATTGAATATGATCGGAATTGTC. The PCR products were resolved by agarose gel electrophoresis. Allele sizes were 499 bp for the wild type and 274 bp for the *Pcyox1* knockout alleles.

### Blood collection and analyses

EDTA-anticoagulated blood samples were obtained by right ventricular puncture in anesthetized mice before euthanasia. Plasma was isolated by centrifugation at 3000 × *g* for 20 min and stored in aliquots at −80 °C. Concentrations of total and free cholesterol, triglycerides, phospholipids, and glucose were determined using enzymatic colorimetric assays (Wako Chemicals). Concentrations of cholesterol esters were calculated by subtracting free cholesterol from total cholesterol. Concentrations of free fatty acids, ALT, and AST were determined using enzymatic colorimetric assays (BioVision Incorporated) according to the manufacturer’s instructions. Plasma apoB was measured by ELISA (Mouse ApoB ELISA Kit, Abcam).

### Histological analysis of atherosclerosis

For quantification of atherosclerotic lesion size in cross-sections of the aortic root, the top half of the heart explanted after mice euthanasia was immersed overnight in 10% neutral buffered formalin and embedded in paraffin. Serial sections (4 μm in thickness) were cut through a ≈300-μm segment of the aortic root, where all 3 valve leaflets were present. For each mouse, 4–5 sections (≈60 μm apart) were stained with Mayer’s hematoxylin and eosin. Images were captured with Axioskop 2 plus microscope equipped with an Axiocam color CCD camera (Zeiss) and the atherosclerotic lesion area was quantified by computer image analysis, using the Axiovision LE rel 4.8 software (Zeiss) by a blinded observer. The average from all measured sections for each animal was used to determine lesion size. Stages of atherosclerotic lesions were classified based on histological criteria defined by Virmani et al.^21^. In addition, the percentage of plaque occupied by cholesterol crystal clefts was determined. For quantification of overall atherosclerotic lesion burden by en face analysis, whole aortas of mice maintained on HFD for 12 weeks were fixed in neutral buffered formalin, rinsed in 0.1 mol/L phosphate buffer, and cleaned using a dissecting microscope to remove any adipose tissue, then opened longitudinally to expose the intimal surface and pinned on black wax-coated dissecting trays. Atherosclerotic lesions were quantified using Axiovision morphometry system and expressed as percentage of the total intimal surface^71^.

### Detection of PCYOX1 protein and mRNA in atherosclerotic lesions

To evaluate the expression of PCYOX1 in human atherosclerotic lesions, two different primary antibodies were used: a rabbit polyclonal anti-PCYOX1 raised against a recombinant protein corresponding to amino acids 172–245 of human PCYOX1 (HPA035193; Prestige Antibodies; Sigma), and a mouse mAb raised against amino acids 356–478 of PCYOX1 of human origin (sc-136391; Santa Cruz Biotechnology). For both the IHC stainings, FFPE sections were subjected to heat-mediated antigen retrieval (HIER) using Dako target retrieval citrate buffer, pH 6.0, prior to commencing immunostaining procedure. Sections were treated with 3% H_2_O_2_ for 10 min to inactivate the endogenous peroxidase and were blocked for non-specific staining by using Background Sniper reagent (Biocare Medical). Sections were then incubated overnight at 4 °C with primary antibody (1:250 in Da Vinci Green antibody diluent, Biocare Medical), followed by incubation with MACH 2 Universal HRP-Polymer (Biocare Medical) for 30 min at room temperature and detection with ImmPACT DAB Peroxidase Substrate (Vector Laboratories). Negative controls were prepared by the omission of primary antibody or using an isotype control antibody (Santa Cruz Biotechnology). At the end of the immunostaining procedure, the sections were counterstained with Mayer’s hematoxylin, dehydrated through ascending alcohols, cleared in xylene, and mounted. Slides were subsequently inspected in bright-field microscopy using an Axioskop 2 plus microscope (Zeiss), and digital images of the selected regions of interest were acquired using Axiovision image analysis system. The same antibodies were also tested on mouse sections, utilizing a rat-on-mouse or mouse-on-mouse HRP polymer detection system (Biocare Medical) as appropriate, according to the manufacturer’s instructions.

Regrettably, commercially available PCYOX1 antibodies did not give reliable IHC results due to either absence of signal or unspecific staining. The rabbit polyclonal antibody HPA035193, which recognizes a portion of the protein encoded by a region of *PCYOX1* gene outside the exon 6, demonstrated immunoreactivity in both Pcyox1^+/+^/Apoe^−/−^ and Pcyox1^−/−^/Apoe^−/−^ mice, likely corresponding to a putative truncated inactive form of the protein. On the other hand, PCYOX1 antibody sc-136391, raised against an AA sequence encoded by exon 6, was not able to detect PCYOX1 protein in mouse sections. Similarly, other commercially available PCYOX1 antibodies did not give reliable IHC results due to either absence of signal or unspecific staining.

Chromogenic ISH for the detection of Pcyox1 transcript in mouse and human atherosclerotic lesions was performed on FFPE sections using an RNAscope assay with RNAscope Probe for murine *Pcyox1* (Mm-Pcyox1-O2; Advanced Cell Diagnostics, ACD, catalog 526511) or human *PCYOX1* (RNAscope Probe Hs-PCYOX1; ACD, catalog 533651) and an RNAscope 2.5 HD Detection Kit (RED) (ACD, catalog 322350) following the manufacturer’s protocols. Both positive (Mm-Ppib and Hs-UBC) and negative (DapB) control probes were included in the procedure according to the manufacturer’s instruction. Nuclei were counterstained with 50% Gill’s Hematoxylin I, allowed to air dry, and mounted in EcoMount mounting medium (Biocare Medical). Slides were subsequently inspected in bright-field microscopy using an Axioskop 2 plus microscope (Zeiss), and digital images of the selected regions of interest were acquired using a ×20 or ×40 objective.

### IHC analysis of human atherosclerotic lesion

For double immunofluorescence labeling of PCYOX1 and apolipoprotein B, FFPE human tissue sections were processed as described for IHC until HIER. Slides were then labeled with the rabbit polyclonal Prestige antibodies against human PCYOX1 (HPA035193) and detected with the Alexa Fluor 594 Tyramide SuperBoost Kit, goat anti-rabbit IgG (Invitrogen; Cat. No. B40944) according to the manufacturer’s instruction. Slides were incubated with 3% H_2_O_2_ for 60 min at room temperature to quench the endogenous peroxidase activity, rinsed with PBS, and blocked for non-specific staining with 10% goat serum. The primary antibody (1:50 dilution in Da Vinci Green antibody diluent) was then applied overnight at 4 °C. Slides were then washed and incubated with poly-HRP-conjugated goat anti-rabbit secondary antibody for 60 min at room temperature, followed by Alexa Fluor tyramide working solution for 10 min and by reaction stop reagent for 15 s. Slides were then rinsed with PBS, incubated with 10% donkey serum for 60 min at room temperature, and labeled with a mouse mAb directed toward the N-terminal amino acids 97–526 of human apoB-100 (apoB (C1.4), sc-13538; Santa Cruz Biotechnology; 1:100 dilution in Da Vinci Green antibody diluent) for 60 min at room temperature. Slides were then washed and detected with a donkey anti-mouse IgG H&L Alexa Fluor 488 secondary antibody (Invitrogen, 1:250) applied for 1 h at room temperature, followed by DAPI counterstain. Images were taken on a confocal microscope (Zeiss LSM710).

Co-staining of PYOX1 and apolipoprotein B in human atherosclerotic lesions was also performed by dual IHC using a multiplex micro-polymer detection kit. Briefly, slides were subjected to HIER using Dako target retrieval citrate buffer (pH 6.0) prior to commencing immunostaining procedure. Sections were then treated with 3% H_2_O_2_ for 10 min to inactivate the endogenous peroxidase, followed by incubation with Background Sniper reagent (Biocare Medical) to block for non-specific staining. Sections were incubated overnight at 4 °C with the primary antibody cocktail (rabbit polyclonal anti-PCYOX1 HPA035193; Prestige Antibodies, Sigma; mouse monoclonal anti-apoB-100 sc-13538; Santa Cruz Biotechnology) diluted 1:250 in Da Vinci Green antibody diluent (Biocare Medical). Detection was performed by incubation with the micro-polymer detection reagent MACH 2 Double Stain 1 (Biocare Medical) for 30 min at room temperature, followed by sequential incubation with ImmPACT DAB Peroxidase Substrate (Vector Laboratories) and Warp Red (Biocare Medical). Adjacent sections were incubated with PCYOX1 or apolipoprotein B primary antibody alone. Negative controls were prepared by the omission of both primary antibodies. At the end of the immunostaining procedure, the sections were counterstained with Mayer’s hematoxylin, dehydrated through ascending alcohols, cleared in xylene, and mounted. Slides were visualized in bright-field microscopy using an Axioskop 2 plus microscope (Zeiss) and images were acquired using an Axiovision image analysis system.

For immunostaining of farnesyl groups deriving from isoprenylated proteins, FFPE human tissue sections were then labeled with the rabbit anti-farnesyl polyclonal antibody (AB4073, 1:200 in Dako diluent, Chemicon International) following the same protocol described for PCYOX1 detection.

### IHC analysis of murine atherosclerotic lesion composition and inflammation

To characterize the composition of aortic root lesions, IHC analysis was performed. FFPE aortic root sections were stained IHC for macrophages (rat monoclonal anti-mouse F4/80 antibody MCA497GA, clone Cl:A3-1, Bio-Rad; 10 µg/mL), neutrophils (rat monoclonal anti-mouse Ly-6B.2 alloantigen, clone 7/4, Bio-Rad; 5 µg/mL), and SMCs (mouse monoclonal anti-alpha smooth muscle actin [α-SMA] antibody, clone 1A4, Sigma; 1:1600 dilution). In addition, to assess the expression of NLRP3 protein, sections were stained with a rabbit polyclonal anti-NLRP3 antibody (ab214185, Abcam; 1 µg/mL). For F4/80 and NLRP3 immunostaining, sections were subjected to HIER using Dako target retrieval citrate buffer, pH 6.0 prior to commencing immunostaining procedure. Sections were treated with 3% H_2_O_2_ to inactivate the endogenous peroxidase and were blocked for non-specific staining by using Rodent Block M reagent (Biocare Medical). Sections were then incubated for 45 min at room temperature or overnight at 4 °C with primary antibody, followed by detection with rat-on-mouse, rabbit-on-rodent, or mouse-on-mouse HRP polymer detection systems (Biocare Medical) as appropriate, according to the manufacturer’s instructions. Negative controls were prepared by the omission of primary antibodies. At the end of the immunostaining procedure, the sections were counterstained with Mayer’s hematoxylin, dehydrated through ascending alcohols, cleared in xylene, and mounted.

Masson’s Trichrome Staining Kit (Bio-Optica) was used to identify collagen within the aortic root sections. Sections were placed in Weigert’s iron hematoxylin solution for 10 min, followed by picric acid alcoholic solution for 4 min. Sections were then rinsed and placed in Ponceau acid fuchsin solution for another 4 min, washed in distilled water, and then placed in phosphomolybdic acid solution for 10 min. Slides were transferred directly to aniline blue solution for 5 min and rinsed in distilled water. Stained sections were dehydrated through ascending alcohols, cleared in xylene, and mounted.

At the end of immunochemical and histological procedures, sections were digitally captured and positive staining was quantified with the Axiovision image analysis software (Zeiss). For each set of stainings, the image that better displayed the full spectrum of color intensity was chosen and used to set the threshold by color sampling. Morphometric analysis of immunopositive areas was then performed by two independent observers in a blinded fashion. To derive an overall measure of lesion vulnerability, VPI was calculated, defined as the ratio of percentage of plaque area occupied by macrophages + extracellular lipids to the percentage of plaque area occupied by SMC + collagen fibers^23^.

### Co-staining of *Pcyox1* mRNA and cell-type-specific protein markers

Co-staining of *Pcyox1* mRNA and cell-type-specific protein markers in mouse atherosclerotic lesions was performed by dual chromogenic ISH-IHC assays. First, ISH analysis was performed for *Pcyox1* mRNA using an RNAscope assay with RNAscope 2.5 HD Detection Kit (RED), as described above. Following ISH detection, sections were stained for macrophages (F4/80) or SMCs (αSMA). Briefly, slides were washed for 5 min in water, followed by blocking with Rodent Block M (Biocare Medical) for 30 min. Anti-mouse F4/80 primary antibody (Bio-Rad; 10 µg/mL) or anti-SMA primary antibody (Sigma; 1:1600 dilution) were added and incubated overnight at 4 °C (F4/80) or 45 min at room temperature (αSMA). Slides were then washed three times in TBST and incubated with the appropriate Biocare HRP polymer (Rat-on-Mouse HRP-Polymer or Mouse-on-Mouse HRP-Polymer) at room temperature for 30 min. Detection was then performed with the ImmPACT DAB Peroxidase Substrate (Vector Laboratories). At the end of the immunostaining procedure, the sections were counterstained with 50% Gill’s Hematoxylin I, allowed to air dry, and mounted in EcoMount mounting medium (Biocare Medical). Slides were subsequently inspected in bright-field microscopy using an Axioskop 2 plus microscope (Zeiss), and digital images of selected regions of interest were acquired using a ×63 objective.

### Quantification of lipid peroxidation in plasma and in aortic lesions

Lipid peroxidation in plasma of Pcyox1^+/+^/Apoe^−/−^ and Pcyox1^−/−^/Apoe^−/−^ mice after 8 weeks on HFD was assayed according to the manufacturer’s instructions using the Thiobarbituric Acid Reactive Substances Assay Kit (Cayman Chemicals), which measures levels of MDA, a byproduct of lipid peroxidation. Quantification was performed by fluorometric measurement (ex 530 nm, em 550 nm).

To assess the extent of lipid peroxidation in aortic root plaques, FFPE tissue sections were stained IHC with a rabbit polyclonal to MDA (ab6463, Abcam; 1 µg/mL). Sections were subjected to HIER in Dako target retrieval citrate buffer pH 6.0, treated with 3% H_2_O_2_, and blocked for non-specific staining by using Rodent Block M reagent (Biocare Medical). Sections were then incubated overnight at 4 °C with the primary antibody, followed by detection with rabbit-on-rodent HRP polymer detection system (Biocare Medical) according to the manufacturer’s instructions. Negative controls were prepared by the omission of the primary antibody. Sections were then processed and digitally captured as described above. Positivity to MDA was quantified with Axiovision as described for the other IHC analyses.

### Inflammatory gene expression in macrophages by qRT-PCR

Thioglycollate-elicited mouse peritoneal macrophages were harvested from the peritoneum of 3–4 mice of each genotype fed standard diet^72^. The cells were seeded in 6-well plates (∼5 × 10^5^ cells/well) in Dulbecco’s Modified Eagle’s medium (Gibco) supplemented with 5% fetal bovine serum (Euroclone) and 1% Pen-Strep (Gibco, ThermoFisher Scientific), and allowed to adhere by culturing them for 2 h at 37 °C. Nonadherent cells were then removed by gently washing with PBS, and macrophages were cultured overnight in a fresh medium. Macrophages were then starved for 1 h in Minimum Essential Medium (Gibco) before stimulation with 5 ng/mL LPS from *Escherichia coli* (LPS, Sigma Aldrich) for 2 h. Analysis of the expression of inflammatory genes by qRT-PCR was performed as described above. Data are expressed as fold increase induced by LPS with respect to unstimulated cells for each strain.

### Statistics and reproducibility

Data analysis was performed using GraphPad Prism 5.0 (GraphPad Software Inc., San Diego, CA). All the data sets were tested for normality of distribution and analyzed by using unpaired two-tailed Student’s *t* test or Mann–Whitney test, accordingly. Analysis of variance was performed in case of more than two conditions. ROS generation over time has been analyzed by linear regression. Categorical data were analyzed with Fisher exact test. Statistical significance level was accepted at *p* < 0.05.

The G * Power 3.1 software was used for sample size calculation in animal studies for approval by Italian Ministry of Health imposing a minimum significance value of 5% and a power of 80%, considering the employed statistical test. For quantification of atherosclerotic lesion size in mice, we established a priori to exclude animals in the following cases: (a) poor quality of aortic root sections (not allowing a reliable evaluation of lesion size); (b) absence of a detectable lesion. For all quantitative parameters, data points were excluded when detected as a significant outlier (based on GraphPad Outlier calculator).

### Reporting summary

Further information on research design is available in the Nature Research Reporting Summary linked to this article.

## Supplementary information

Supplemental information file

Description of Additional Supplementary Files

Supplementary Data 1

Reporting Summary

## Data Availability

Data collected in the study will be made available using the data repository Zenodo (https://zenodo.org/; 10.5281/zenodo.5235364). Proteomic data are available via ProteomeXchange with identifier PXD022302 and 10.6019/PXD022302. Any remaining information can be obtained from the corresponding author upon reasonable request.

## Materials availability

Further information and requests for resources and reagents should be directed to and will be fulfilled by the lead contact, Cristina Banfi (cristina.banfi@ccfm.it). The mass spectrometry proteomics data have been deposited to the ProteomeXchange Consortium via the PRIDE^73^ partner repository with the dataset identifier PXD022302 and 10.6019/PXD02230.
