# Supplementary material for: Prenylcysteine oxidase 1, an emerging player in atherosclerosis
Source: Commun Biol. 2021 Sep 21;4:1109. doi: 10.1038/s42003-021-02630-z (PMC8455616; doi:10.1038/s42003-021-02630-z)
Supplement: Supplementary file 1 — Supplemental information file [file 42003_2021_2630_MOESM1_ESM.pdf]

Prenylcysteine oxidase 1, an emerging player in atherosclerosis

C. Banfi[1*], R. Baetta, [1], S. S. Barbieri [1], M. Brioschi [1], A. Guarino [2], S. Ghilardi [1], L. Sandrini [1], S. Eligini[1], G. Polvani [2,3,4], O. Bergman [5], P. Eriksson [5], E. Tremoli [1].

[1] Centro Cardiologico Monzino IRCCS, 20138 Milano, Italy

[2] Cardiovascular Tissue Bank of Milan, Centro Cardiologico Monzino IRCCS, 20138 Milano, Italy

[3] Department of Clinical Sciences and Community Health, Cardiovascular Section, University of Milan, 20138 Milano, Italy

[4] Department of Cardiovascular Disease, Development and Innovation Cardiac Surgery Unit, Centro Cardiologico Monzino IRCCS, 20138 Milano, Italy

[5] Department of Medicine Solna, Karolinska University hospital, Karolinska Institutet, 171 76 Stockholm, Sweden.

*corresponding author

Corresponding author

Cristina Banfi, PhD

Head, Unit of Proteomics

Monzino Cardiologic Center IRCCS

20138, via Parea, 4

phone: +39-0258002403

FAX: +39-0258002623

Email: cristina.banfi@ccfm.it

SUPPLEMENTARY MATERIAL

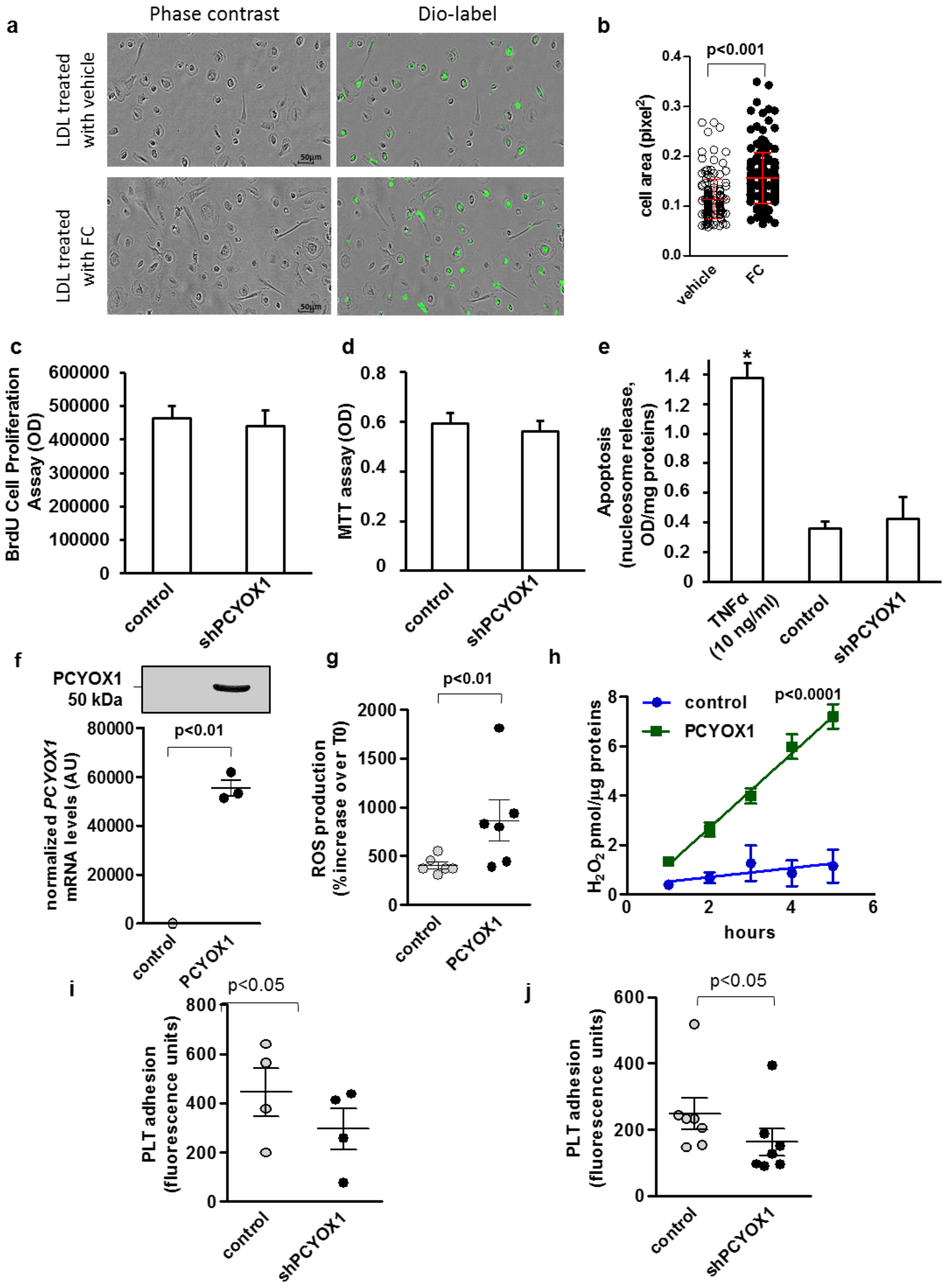

Supplementary Figure 1. Human macrophages were incubated with 50 μg/ml of vehicle-treated LDL or FC-treated LDL, both labelled with 3,3′-Dioctadecyloxacarbocyanine perchlorate (Dio). Representative images acquired with Incucyte in phase contrast or for green fluorescence (a) and measurement of cell area obtained with ImageJ software (b). Data are presented as circle-plot, with each circle representing an individual sample and bars showing the mean value±SEM, $p<0.001$ by Student's t test. c-e) Effects of PCYOX1 silencing on HepG2. c) Cell proliferation assayed by BrdU incorporation. Values are the means±SEM of 4 individual experiments. d) MTT assay. Values are the means±SEM of 4 individual experiments. e) Cell apoptosis assay. TNFα (10 ng/ml) was used as positive control. The data are expressed as absorbance/mg of cell proteins as determined by the Bradford protein assay. Values are the means±SD of 4 individual experiments. *$p<0.01$ vs control cells by Student's t test. f) *PCYOX1* mRNA normalized to the housekeeping gene 18S rRNA (n=3), protein (n=3) and intracellular ROS production (n=6) (g) in control or in PCYOX1-overexpressing CHO cells. $p<0.01$ vs control cells by Student's t test. Data are presented as circle-plot, with each circle representing an individual sample and bars showing the mean value±SEM. h) H_2O_2 produced by CHO cell lysates in the presence of FC, means±SEM, n=3. Statistical significance was calculated by linear regression, $p<0.0001$. i-j) PCYOX1 silencing reduces the adhesion of human platelets to fibrinogen and endothelial cells. i) Washed human platelets, stained with calcein, were allowed to adhere to immobilized fibrinogen for 30 min in the presence of the secretome from control and PCYOX1-silenced cells. Data are expressed as fluorescence intensity after cell lysis from 4 independent experiments, means±SEM. j) Platelet adhesion to endothelial cells (HUVEC) was quantified based on the fluorescence detection of labelled platelets. $p<0.05$ vs control cells by Student's t test. Data are presented as circle-plot, with each circle representing an individual sample and bars showing the mean value±SEM. Related to Figure 2.

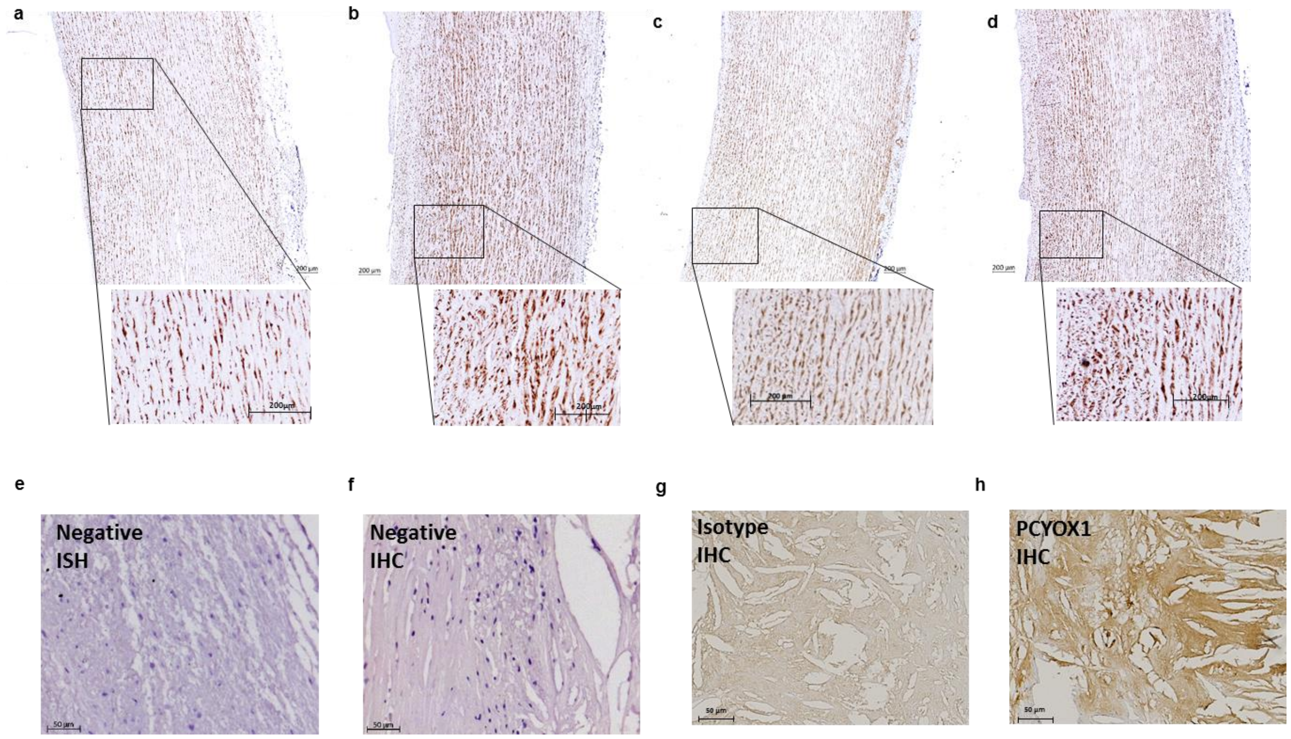

Supplementary Figure 2. a-d) Normal human arterial tissue exhibits elevated expression of PCYOX1 in medial SMCs. Formalin-fixed paraffin-embedded sections from normal human arteries were subjected to IHC for the detection of PCYOX1 protein. Primary antibody (sc-136391) was detected as DAB reaction product (brown staining) with a MACH 2 Universal HRP-Polymer (Biocare Medical). Tissue sections were counterstained with hematoxylin. Shown are tissue sections obtained from 2 female subjects (a and b; age: 46 and 52 years, respectively) and 2 male subjects (c and d; age: 25 and 53 years, respectively). e-g) Representative images of negative controls for PCYOX1 staining in the human atherosclerotic lesion. e) Negative control for PCYOX1 in situ hybridization (DapB probe). f) Negative control for PCYOX1 immunostaining obtained without the primary antibody. g) Immunostaining with isotype antibody. h) Immunostaining with PCYOX1 antibody. All images refer to tissue sections from the same donor (Objective 20x). Related to Figure 3.

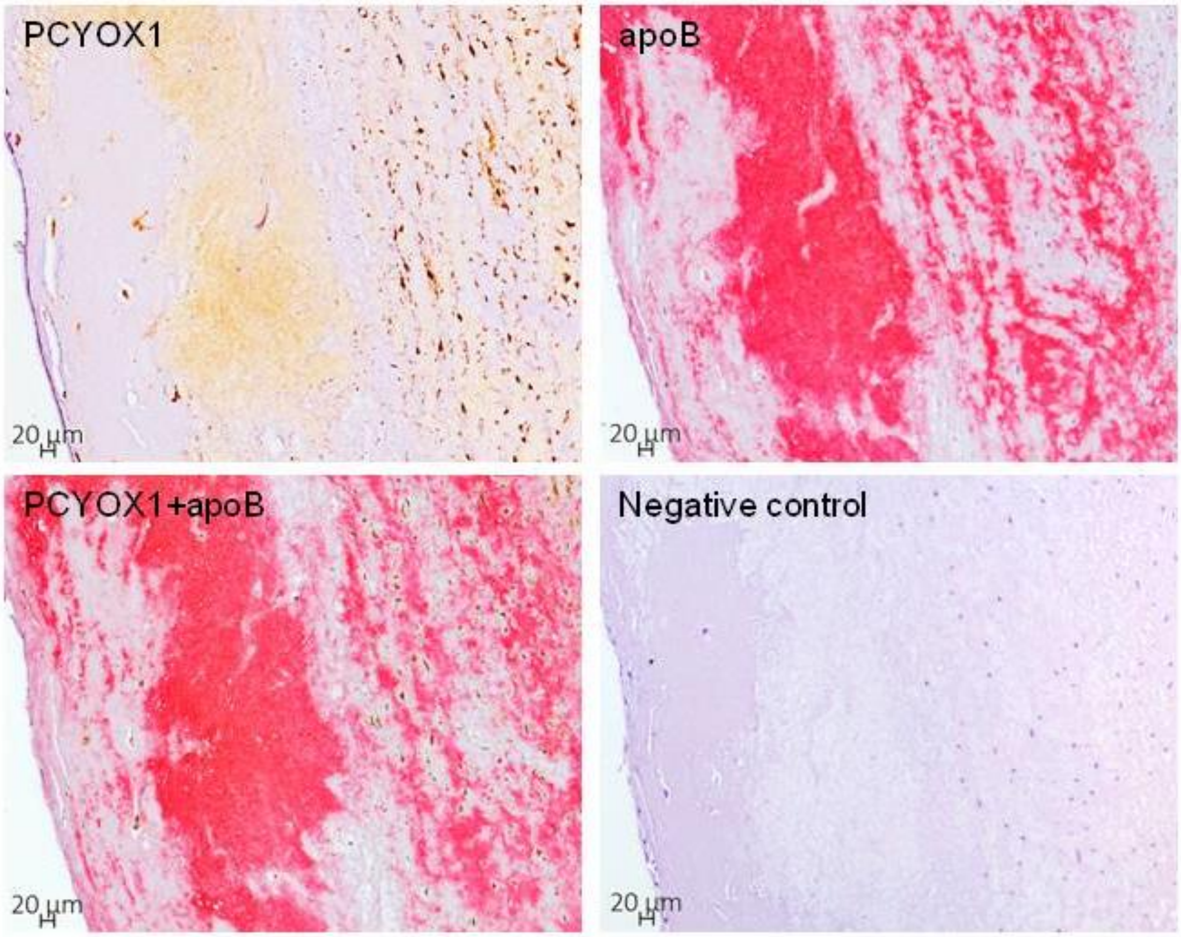

Supplementary Figure 3. Extracellular staining for PCYOX1 in human atherosclerotic lesions displays substantial overlapping with apolipoprotein B deposits. Formalin-fixed paraffin-embedded sections of human atherosclerotic lesions were subjected to dual IHC for simultaneous detection of PCYOX1 and apolipoprotein B using a MACH 2 Double Stain 1 micro-polymer detection system (Biocare Medical). Rabbit polyclonal anti-PCYOX1 HPA035193 antibody was detected as DAB reaction product (brown staining) and mouse monoclonal anti-apoB-100 antibody was detected as Biocare's Warp Red reaction product (bright fuchsin-red staining). Shown are representative images from serial sections immunostained for PCYOX1 and apoB, alone or in combination, or incubated in the absence of primary antibodies (negative control). Related to Figure 3.

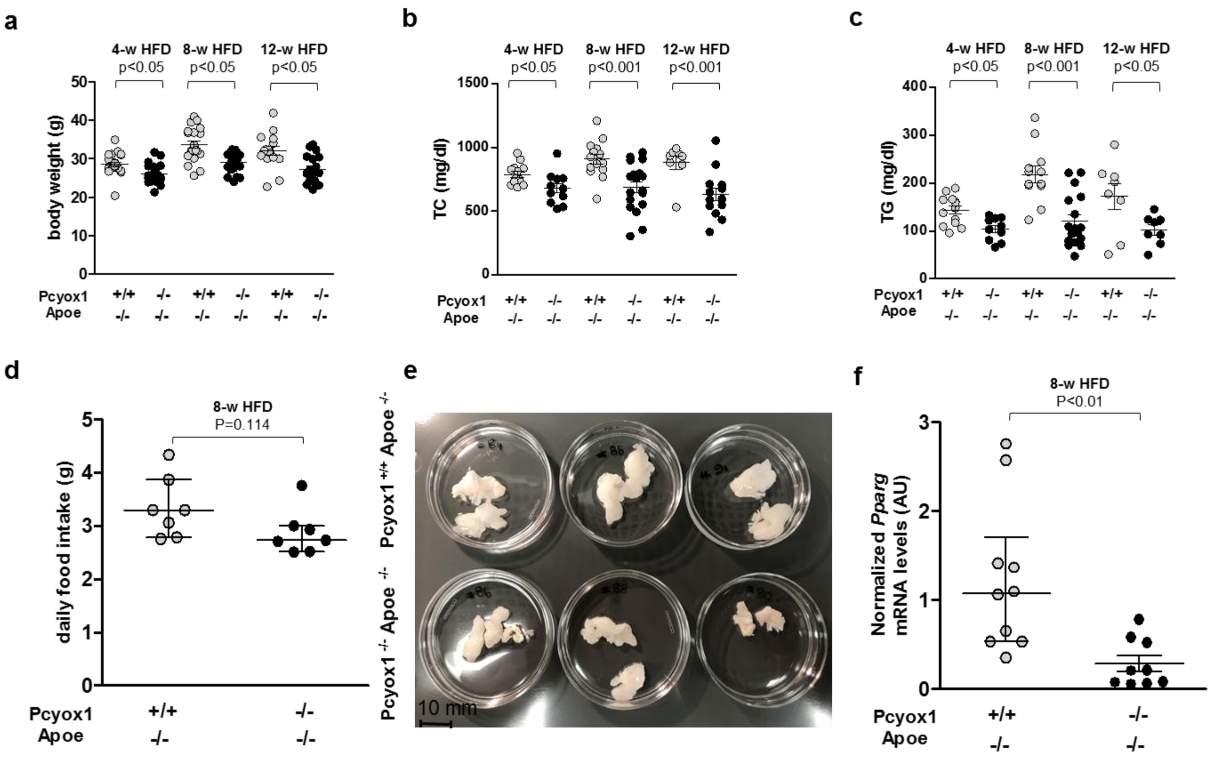

Supplementary Figure 4. PCYOX1 deficiency effects on plasma lipids, food intake, adipose tissue depots and liver metabolism gene expression. a-c) Body weight and plasma lipids at different time points. Daily food intake (d) and representative images of adipose tissues (e) from Pcyox1$^{-/-}$/Apoe $^{-/-}$ and Pcyox1 $^{-/-}$ /Apoe $^{-/-}$ fed for 8 weeks with a high-fat diet (HFD). f) *Pparg* mRNA levels normalized to the housekeeping gene *Gapdh* evaluated in the liver of Pcyox1$^{-/-}$ /Apoe $^{-/-}$ and Pcyox1 $^{-/-}$ $^{/}$Apoe $^{-/-}$ fed for 8 weeks with a HFD. p values have been calculated by Student's t test. Data are presented as circle-plot, with each circle representing an individual sample and bars showing the mean value±SEM. Related to Figure 4.

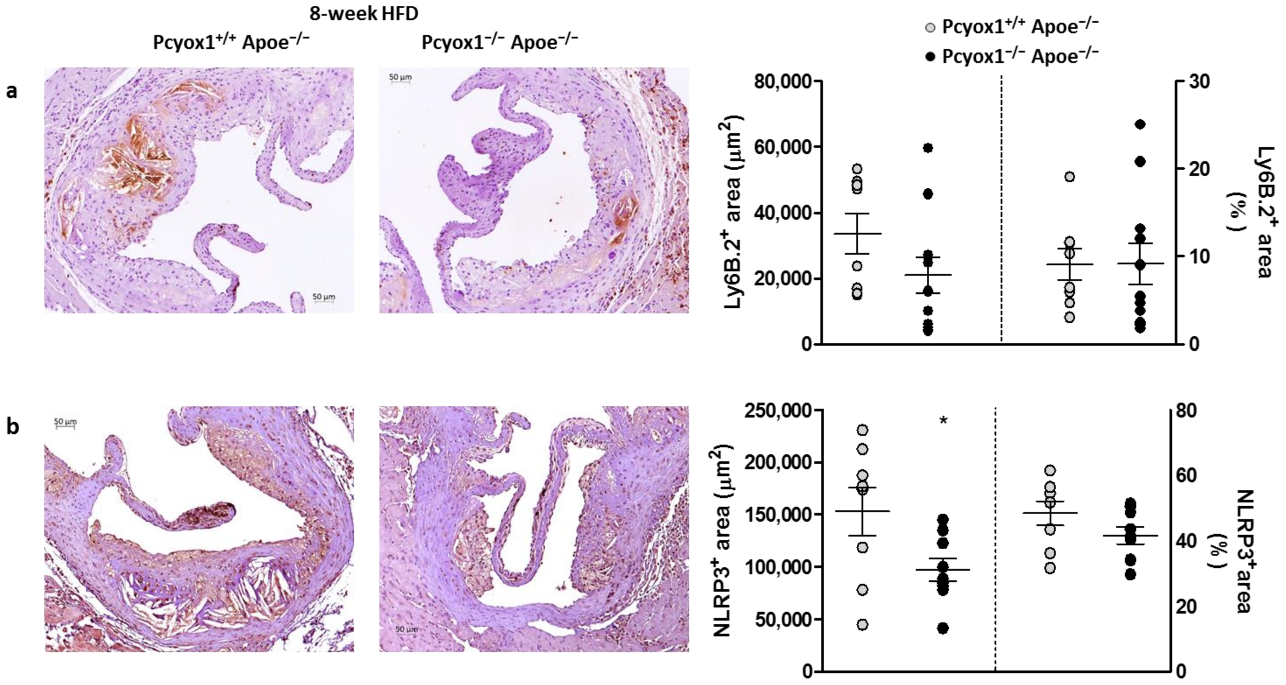

Supplementary Figure 5. Pcyox1 deficiency is associated with a minor content of neutrophils and a lower expression of NLRP3 protein. Morphometric analysis of atherosclerotic lesions from aortic roots of Pcyox1$^{+/+}$/Apoe$^{-/-}$ (n=8, panel a; n=8 panel b) and Pcyox1$^{-/-}$ /Apoe$^{-/-}$ (n=11, panel a; n=9 panel b) mice fed a HFD for 8 weeks to assess the effect of Pcyox1 deletion on neutrophil content (a) and NLRP3 expression (b). Representative images and quantification of: a, neutrophil content (Ly-6B.2 staining); b, inflammasome (NLRP3 staining). Shown on the right are the results of the morphometric analysis for the stainings, expressed either as absolute positive areas or as percentage of total plaque area; data are presented as circle-plot, with each circle representing an individual sample and bars showing the mean value±SEM. * $p<0.05$ by Student’s t test. Related to Figure 6.

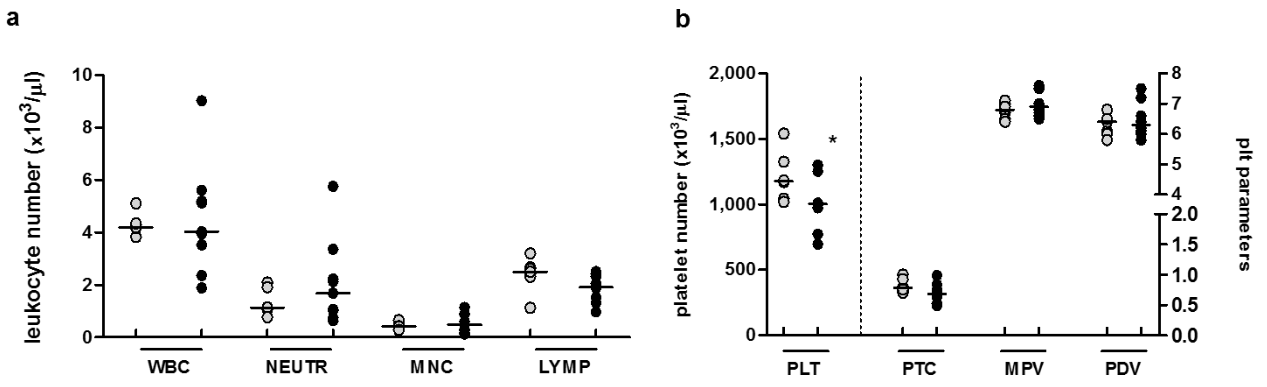

Supplementary Figure 6. Pcyox1 deficiency does not affect blood leukocyte counts but is associated with a decreased number of circulating platelets. EDTA-anticoagulated blood samples from Pcyox1$^{+/+}$ /Apoe$^{-/-}$ (grey circles) and Pcyox1$^{-/-}$ /Apoe$^{-/-}$ (black circles) mice fed an HFD for 8 weeks were subjected to hematologic analysis to assess the effect of Pcyox1 deletion on leukocyte and platelet count. The analysis showed that the total leukocyte count (WBC), and the cell count of the main leukocyte subsets (polymorphonuclear neutrophils, NEUTR; monocytes MNC; and lymphocytes, LYMP) were comparable in the two genotypes (a). In contrast, platelet number (PLT) in Pcyox1$^{-/-}$/Apoe$^{-/-}$ mice was significantly lower in comparison to Pcyox1$^{+/+}$/Apoe$^{-/-}$ control mice, with no changes in plateletcrit (PTC), mean platelet volume (MPV) and platelet distribution width (PDV) (b).* $p<0.05$ by Student's t test, Bars indicate the median.

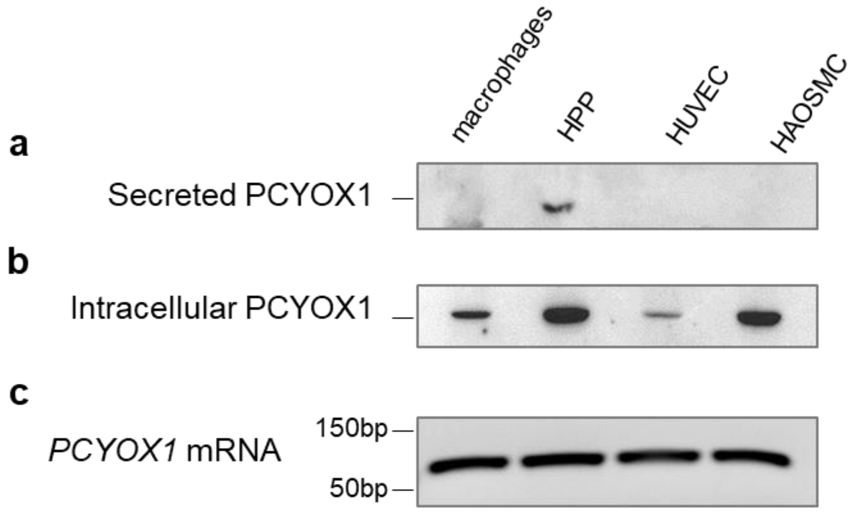

Supplementary Figure 7. *PCYOX1* mRNA, intracellular protein level, and PCYOX1 secretion in human macrophages, human primary hepatocytes (HPP), human endothelial cells (HUVEC), and smooth muscle cells (HAOSMC). a and b, an equal amount of proteins were separated by SDS-PAGE and immunoblotted with an antibody against human PCYOX1. c, mRNA levels of *PCYOX1* analysed by RT-PCR.

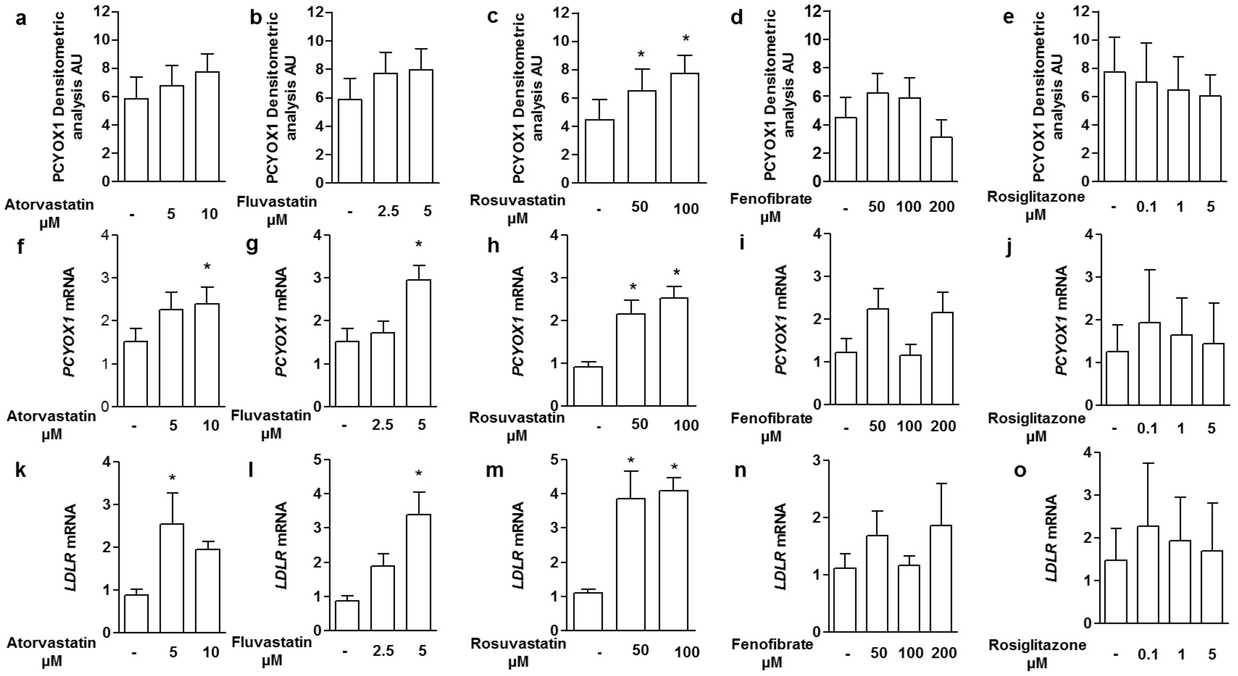

Supplementary Figure 8. Pharmacological modulation of PCYOX1 expression in HepG2 cells by HMG-CoA inhibitors, fenofibrate and rosiglitazone. HepG2 cells were treated for 48 hours in complete medium containing drugs or vehicle, and PCYOX1 levels were evaluated by immunoblotting at protein level (Atorvastatin, a; Fluvastatin, b; Rosuvastatin, c; Fenofibrate, d; Rosiglitazone, e) and real-time PCR at mRNA level (Atorvastatin, f; Fluvastatin, g; Rosuvastatin, h; Fenofibrate, i; Rosiglitazone, j). 18s mRNA has been used as the housekeeping gene for real-time PCR. Data are representative of 3 independent experiments. * $p<0.05$ vs vehicle–treated cells by Student's t test. Pharmacological modulation of LDL receptor (LDLR) expression in HepG2 cells by HMG-CoA inhibitors, fenofibrate and rosiglitazone. HepG2 cells were treated for 48 hours in complete medium containing drugs or vehicle, and *LDLR* levels were evaluated by real-time PCR (Atorvastatin, k; Fluvastatin, l; Rosuvastatin, m; Fenofibrate, n; Rosiglitazone, o). 18s mRNA has been used as housekeeping gene for real-time PCR. Data are representative of 3 independent experiments with bars showing the mean value±SEM. * $p<0.05$ vs vehicle-treated cells by Student's t test.

Supplementary Table 1. List of the secreted proteins modulated by PCYOX1 silencing in HepG2 cells identified by means of a label-free mass spectrometry-based proteomic approach, LC-MS^E. Data have been generated from four independent experiments performed in triplicate. Related to Figure 2.

Accession	Description	Highest mean condition	# Peptides[1]	Score	p value	Max fold change
Proteins less abundant after PCYOX1 silencing						
P07996	Thrombospondin-1, THBS1	control	24/20	154.2	6.69E-11	1.64
P02768	Serum albumin, ALB	control	68/68	702.1	0	1.73
Q08830	Fibrinogen-like protein 1, FGL-1	control	8/8	47.0	4.61E-07	1.41
P01033	Metalloproteinase inhibitor 1, TIMP1	control	2/2	13.4	0.058383	1.44
P08833	Insulin-like growth factor-binding protein 1, IGFBP1	control	14/14	125.6	3.36E-07	1.49
P01011	Alpha-1-antichymotrypsin, AACT	control	17/15	148.2	0.000252	1.35
Q99988	Growth/differentiation factor 15, GDF15	control	5/5	35.8	2.29E-07	1.35
P10646	Tissue factor pathway inhibitor, TFPI	control	10/10	81.8	2.46E-07	1.39
P02766	Transthyretin, TTR	control	8/8	75.0	0.073035	1.31
P61626	Lysozyme C, LYZ	control	11/9	91.0	0.000168	1.30
P05121	Plasminogen activator inhibitor 1, SERPINE1	control	11/11	78.2	0.045464	1.23
P01023	Alpha-2-macroglobulin, A2M	control	83/70	836.1	3.14E-05	1.25
P01024	Complement C3, C3	control	102/98	983.0	2.06E-06	1.19
P29279	Connective tissue growth factor, CTGF	control	14/12	112.8	2.75E-05	1.27
P19883	Follistatin, FST	control	9/9	56.2	0.000876	1.25
Proteins more abundant after PCYOX1 silencing						
P08238	Heat shock protein HSP 90-beta, HSP90AB	shPCYOX1	7/3	43.4	0.002578	1.74
P07437	Tubulin beta chain, TUBB	shPCYOX1	5/4	30.0	0.000143	1.60
P68104	Elongation factor 1-alpha 1, EEF1A1	shPCYOX1	4/4	26.8	4.81E-07	1.54
P07900	Heat shock protein HSP 90-alpha, HSP90AA1	shPCYOX1	7/3	42.6	0.000705	1.61
P04406	Glyceraldehyde-3-phosphate dehydrogenase, GAPDH	shPCYOX1	4/3	27.3	2.53E-05	1.50
P11142	Heat shock cognate 71 kDa protein, HSP8A	shPCYOX1	7/2	47.8	8.97E-07	1.38
P60709	Actin, cytoplasmic 1, ACTB	shPCYOX1	13/2	116.2	0.000819	1.29
P14618	Pyruvate kinase, PKM	shPCYOX1	5/3	26.7	0.000166	1.26
P07148	Fatty acid-binding protein, liver , FABP1	shPCYOX1	4/3	26.6	0.000125	1.26
P00338	L-lactate dehydrogenase A chain, LDHA	shPCYOX1	6/5	40.5	0.000454	1.24
P60174	Triosephosphate isomerase, TPI1	shPCYOX1	10/8	62.7	0.008772	1.20
P01034	Cystatin-C, CST3	shPCYOX1	8/8	53.6	6.97E-06	1.25
P62987	Ubiquitin-60S ribosomal protein L40, UBA52	shPCYOX1	3/3	22.4	0.026215	1.23

P00558	Phosphoglycerate kinase 1, PGK1	shPCYOX1	2/2	11.4	0.006522	1.27

[1] Peptide count / Peptides used for quantitation

Supplementary Table 2. List of the secreted proteins modulated by PCYOX1 silencing in HepG2 cells identified by means of multiplex immunoassay. Related to figure 2.

Uniprot accession	Protein description	Fold shPCYOX1/control
Proteins more abundant after PCYOX1 silencing		
P13726	Tissue factor (TF)	2.05
P04080	Cystatin-B (CSTB)	2.02
P12931	Proto-oncogene tyrosine-protein kinase Src (SRC)	1.99
Q14790	Caspase-8 (CASP-8)	1.88
Q9Y6K9	NF-kappa-B essential modulator (NEMO)	1.75
P35318	Adrenomedullin (AM)	1.60
P25116	Proteinase-activated receptor 1 (PAR-1)	1.50
P09341	C-X-C motif chemokine 1 (CXCL1)	1.46
P07711	Cathepsin L1 (CTSL1)	1.43
O14763	TNF-related apoptosis-inducing ligand receptor 2 (TRAIL-R2)	1.39
P09603	Macrophage colony-stimulating factor 1 (CSF-1)	1.35
P25445	Tumor necrosis factor receptor superfamily member 6 (FAS)	1.31
O00253	Agouti-related protein (AGRP)	1.27
Proteins less abundant after PCYOX1 silencing		
P10145	Interleukin-8 (CXCL8)	0.83
P19883	Follistatin (FST)	0.73
Q9H2A7	C-X-C motif chemokine 16 (CXCL16)	0.72
Q99988	Growth/differentiation factor 15 (GDF-15)	0.69
P09237	Matrix metalloproteinase-7 (MMP-7)	0.58
Q03405	Urokinase plasminogen activator surface receptor (PLAUR)	0.52
O94907	Dickkopf-related protein 1 (DKK-1)	0.34

Supplementary Table 3. Body weight, plasma lipids levels and glucose from $Pcyox1^{+/+}/Apoe^{-/-}$ and $Pcyox1^{-/-}/Apoe^{-/-}$ mice fed a chow diet, and age-matched with mice fed HFD for 8 weeks.

Measurement	$Pcyox1^{+/+}/Apoe^{-/-}$	$Pcyox1^{-/-}/Apoe^{-/-}$	*P*
Body weight, g	30.7 ± 2.4 (10)	27.93 ± 2.2 (6)	<0.05
Total cholesterol, mg/dL	300 ± 69 (10)	279 ± 67 (6)	n.s.
Free cholesterol, mg/dL	124 ± 23 (10)	119 ± 23 (6)	n.s.
Cholesterol esters, mg/dL	176 ± 50 (10)	161 ± 46 (6)	n.s.
Triglycerides, mg/dL	91 ± 19 (10)	75 ± 27 (6)	n.s.
Phospholipids, mg/dL	199 ± 31 (10)	180 ± 40 (6)	n.s.
Glucose, mg/dL	196 ± 50 (10)	214 ± 86 (6)	n.s.

Mean ± SD
