# Supplementary material for: Prenylcysteine oxidase 1, an emerging player in atherosclerosis
Source: Commun Biol. 2021 Sep 21;4:1109. doi: 10.1038/s42003-021-02630-z (PMC8455616; doi:10.1038/s42003-021-02630-z)
Supplement: Supplementary file 2 — Description of Additional Supplementary Files [file 42003_2021_2630_MOESM2_ESM.pdf]

Description of Additional Supplementary Files

File name: Supplementary Data 1.

Description: Functional enrichments in the network created by STRING with secreted proteins modulated by PCYOX1 silencing. Related to Figure 2.
